# Dysregulated proteasome activity and steroid hormone biosynthesis are associated with mortality among patients with acute COVID-19

**DOI:** 10.1186/s12967-024-05342-0

**Published:** 2024-07-04

**Authors:** Fengjiao Liu, Huqin Yang, Tingyu Yang, Zhijin Zhang, Lujia Guan, Leyi Gao, Haomiao Ma, Haifan Zhang, Nan Song, Zhaohui Tong, Jieqiong Li

**Affiliations:** 1 Department of Respiratory and Critical Care Medicine, Beijing Institute of Respiratory Medicine, Beijing Chao-Yang Hospital, Capital Medical University, 8 Workers Stadium South Road, Chaoyang District, Beijing, China; 2 Medical Research Center, Beijing Institute of Respiratory Medicine and Beijing Chao-Yang Hospital, Capital Medical University, Beijing, China

**Keywords:** COVID-19, Omics, Acute phase, Mortality, Prediction

## Abstract

The persistence of coronavirus disease 2019 (COVID-19)-related hospitalization severely threatens medical systems worldwide and has increased the need for reliable detection of acute status and prediction of mortality. We applied a systems biology approach to discover acute-stage biomarkers that could predict mortality. A total 247 plasma samples were collected from 103 COVID-19 (52 surviving COVID-19 patients and 51 COVID-19 patients with mortality), 51 patients with other infectious diseases (IDCs) and 41 healthy controls (HCs). Paired plasma samples were obtained from survival COVID-19 patients within 1 day after hospital admission and 1–3 days before discharge. There were clear differences between COVID-19 patients and controls, as well as substantial differences between the acute and recovery phases of COVID-19. Samples from patients in the acute phase showed suppressed immunity and decreased steroid hormone biosynthesis, as well as elevated inflammation and proteasome activation. These findings were validated by enzyme-linked immunosorbent assays and metabolomic analyses in a larger cohort. Moreover, excessive proteasome activity was a prominent signature in the acute phase among patients with mortality, indicating that it may be a key cause of poor prognosis. Based on these features, we constructed a machine learning panel, including four proteins [C-reactive protein (CRP), proteasome subunit alpha type (PSMA)1, PSMA7, and proteasome subunit beta type (PSMB)1)] and one metabolite (urocortisone), to predict mortality among COVID-19 patients (area under the receiver operating characteristic curve: 0.976) on the first day of hospitalization. Our systematic analysis provides a novel method for the early prediction of mortality in hospitalized COVID-19 patients.

**Supplementary Information:**

The online version contains supplementary material available at 10.1186/s12967-024-05342-0.

## Background

Since 2019, coronavirus disease 2019 (COVID-19), caused by severe acute respiratory syndrome coronavirus 2 (SARS-CoV-2), has emerged as a global public health threat because of its widespread dissemination. By June 2023, there were > 700 million confirmed cases and > 6.9 million deaths; thus, COVID-19 has caused one of the worst pandemics in human history [1]. Although vaccination and antiviral therapies have shown considerable promise, the crude mortality risk among hospitalized COVID-19 patients remains high (4.9%) [1, 2]. Clinical manifestations of SARS-CoV-2 infection substantially vary, ranging from asymptomatic infection to severe disease [3]. Several epidemiological factors are reportedly associated with adverse outcomes, such as male sex, older age, and certain comorbidities [4, 5]. However, these factors only partially explain the broad clinical spectrum of COVID-19 manifestations among affected patients. Thus, there is an urgent need to clarify host factors that contribute to susceptibility to adverse outcomes, or to predict which COVID-19 patients have a high risk of adverse outcomes.

The blood ecological information bank is a complex network of highly coordinated interactions among diverse molecules, including proteins and metabolites. These molecular interactions offer insights concerning the specific characteristics of disease onset and progression. Multi-omics analysis of the blood molecular interaction can provide a complete picture of the pathophysiological landscape. Recent research has shown that, in addition to the involvement of viral factors, disease severity largely depends on host status; thus, it is important to consider molecular responses in each patient [6–9]. Changes in host metabolism and the plasma proteome are presumably involved in viral pathogenesis and multiorgan failure; an understanding of these factors could facilitate the discovery of key factors driving infectious disease progression [9, 10]. Our previous study investigated proteomic and metabolomic changes in community-acquired pneumonia patients and identified a panel of indicator proteins for severe pneumonia [11]. Additionally, we explored immune responses and molecular mechanisms induced by SARS-CoV-2 vaccines using a multi-omic approach [12]. Thus, proteomic and metabolomic analyses have provided comprehensive insights into the pathogenesis of various infectious diseases, including Ebola virus disease, community-acquired pneumonia, and *Staphylococcus aureus* bacteremia [13–15], establishing a foundation for similar studies concerning COVID-19.

Thus far, metabolomic and proteomic analyses have mainly focused on the identification of biomarkers for COVID-19 diagnosis and severity assessment [16–19], rather than specific disease features associated with different disease trajectories among hospitalized patients. Proteomic and metabolomic analyses have revealed the dysregulation of multiple immune factors and metabolites that are correlated with disease severity [16]. Moreover, the IMMuno Phenotyping Assessment in a COVID-19 Cohort study defined the immune and biological states of COVID-19 patients during the first 28 days of hospitalization [20]. Richard et al. combined multi-omic data with a machine learning model to predict outcomes among hospitalized COVID-19 patients, but their study was hindered by a relatively small sample size and lack of disease controls [21]. To our knowledge, there have been few comprehensive and unbiased multi-omic analyses to elucidate dynamic changes across COVID-19 phases, especially concerning factors that can distinguish recovery from deterioration.

The present study assessed changes in host response and defined precise features of disease trajectories, with the goal of establishing a multivariate module for mortality prediction. Accordingly, we integrated proteomic and metabolomic analyses of plasma samples from a cohort of surviving hospitalized COVID-19 patients [acute COVID-19 (COVID-19-A) and recovered COVID-19 (COVID-19-R)], COVID-19 patients with mortality (COVID-19-M), other infectious disease controls (IDCs), and healthy controls (HCs). Machine learning models were constructed to identify specific patterns of COVID-19 and to discover acute-stage biomarkers that could predict mortality; these results were validated by enzyme-linked immunosorbent assays (ELISAs) and metabolomic analyses in an independent cohort. Integrated proteomic and metabolomic analyses further helped to elucidate mechanisms underlying the pathogenesis of COVID-19. Overall, our results can promote progress in screening and treatment strategies for COVID-19.

## Materials and methods

### Study design and patient information

In total, 155 plasma samples were collected from 103 patients with confirmed COVID-19 at Beijing Chao-Yang Hospital. Of these patients, 52 were discharged from the hospital and 51 died. Among the 52 discharged patients, samples were collected in both the acute phase (1 day after admission) and the recovery phase (1–3 days before discharge, Supplementary Data 1.1). Among the 51 COVID-19-M patients, plasma samples were collected at the time of hospitalization (Supplementary Data 1.2). COVID-19 patients were included in this study after implementation of the Diagnosis and Treatment Plan for Novel Coronavirus Infection (Trial Version 10).

Additionally, 51 patients with other infectious diseases (IDCs) were included in the study. These patients had respiratory symptoms and COVID-19-negative results in reverse transcription polymerase chain reaction (RT-PCR) assays (Supplementary Data 1.3). Forty-one healthy volunteers from the period before the SARS-CoV-2 pandemic, with COVID-19-negative results in RT-PCR, were included as the HC group (Supplementary Data 1.4). This study protocol was approved by the Ethics Committee of Beijing Chao-Yang Hospital (2021-KE-500). Informed consent was obtained from all participants.

### Clinical measurements and sample handling

Patients’ electronic medical records were reviewed to collect demographic and clinical information. Metadata variables collected in this study included demographics and clinical laboratory results [white blood cell (WBC), neutrophil (Neu), lymphocyte (Lym), red blood cell (RBC), hemoglobin (Hgb), platelet (Plt), lactic acid (Lac), and oxygenation index (PaO_2_/FiO_2_)]. Disease severity was evaluated using an eight-category ordinal scale after participants had enrolled in the study [22].

### Proteomic data acquisition

In total, 50 samples from 40 participants [10 surviving COVID-19 patients (acute and recovery phases), 10 COVID-19 patients with mortality (COVID-19-M), 10 IDCs, and 10 HCs] were subjected to proteomic analysis as previously described [11, 12]. Each specimen was denatured in 100 µL of buffer (8 M urea in 100 mM triethylammonium bicarbonate) at 25℃ for 30 min. The mixture was reduced with 5 mM Tris phosphine (Pierce, Rockford, IL, USA) and then alkylated using 15 mM iodoacetamide (Sigma-Aldrich, St. Louis, MO, USA). The protein extract was mixed with Trypsin Gold, Mass Spectrometry Grade (Promega, Madison, WI, USA) and digested overnight at 37℃. The resulting peptides were dried and solubilized in 20 µL of loading buffer (1% formic acid and 1% acetonitrile). Ten microliters of sample were analyzed by liquid chromatography-tandem mass spectrometry (LC-MS/MS) in the data-dependent acquisition mode to construct a high-quality library. The peak area obtained from MS1 intensity was used to quantify each peptide.

### ELISA analysis

The levels of selected biomarkers were determined by ELISA in samples from the proteomic cohort (*n* = 40) and an independent cohort (*n* = 155). Inflammation-related proteins [C-reactive protein (CRP), serum amyloid A-1 (SAA1), SAA2, and alpha-1-acid glycoprotein 1 (ORM1)], three immunity-related proteins [immunoglobulin heavy constant gamma 1 (IGHG1), immunoglobulin lambda-like polypeptide 5 (IGLL5), and IGHG3] and four proteasome subunit proteins [proteasome subunit alpha type-1 (PSMA1), PSMA6, PSMA7 and proteasome subunit beta type-1 (PSMB1)] kits were investigated. Protein abundances were determined in accordance with the manufacturer’s instructions.

### Metabolomic data acquisition

All plasma samples were subjected to untargeted metabolomics. 400 µL of Methanol (MeOH)/ acetylcholine (ACH, 1:1, v/v) solvent mixture were added to each 100-µL plasma sample. After incubation and centrifugation, the supernatant was collected and divided into three groups, as previously described [12]. All ultra performace liquid chrmatography-electrospray tandem mass spectrometry (UPLC-MS/MS) methods were performed using an ACQUITY 2D UPLC system (Waters, Milford, MA, USA) and Q Exactive HF hybrid Quadrupole-Orbitrap (Thermo Fisher Scientific, San Jose, USA) with an electrospray ionization source and a C18 column (UPLC BEH C18, 2.1 × 100 mm, 1.7 μm; Waters) in positive and negative mass analyzer modes. The mass range extended from 100 to 1,000 m/z. For full MS scans, the resolution was set to 70,000; for higher-energy collisional dissociation MS/MS scans, the resolution was set to 17,500. Collision energies were set to 10, 20, and 40 eV. Quality controls were injected after every 20th sample to provide a dataset that could be used to assess repeatability throughout the analysis.

### Statistical analysis

Categorical and continuous variables were analyzed by Student’s t-test and the Chi-square test, respectively. Fold changes in proteins and metabolites were calculated using the mean relative abundance across patients in each pair of comparison groups. Two-sided unpaired Welch’s t-tests were used to calculate the statistical significance of differences in proteins and metabolites. We recorded differentially expressed proteins (DEPs) and differentially expressed metabolites (DEMs) with *P*-values < 0.05 and fold changes ≥ 1.5 or < 0.67. *P*-values were adjusted by Benjamini–Hochberg correction (*P* adjust < 0.05). Statistical significance in multigroup analyses were calculated by one-way analysis of variance (ANOVA) and Tukey’s honestly significant difference (HSD) test. Partial least squares discriminant analysis (PLS-DA) was performed for classification using MetaboAnalyst 5.0 (https://www.metaboanalyst.ca/).

To investigate biological processes, Gene Ontology (GO, http://geneontology.org/) and Kyoto Encyclopedia of Genes and Genomes (KEGG) pathway (http://www.genome.jp/kegg/) analyses were conducted based on DEPs and DEMs. To explore dynamic patterns, clustering trends were constructed using the Mfuzz package (version 2.46.0) in R software. For gene set enrichment analysis (GSEA), clusterProfiler was utilized; the Path view package was used to visualize protein-level changes in the indicated pathways.

### Model construction and evaluation

Survival prediction was performed using the survival [3.3.1], survminer, and ggplot2 [3.3.6] packages in R software to establish a machine learning model. Area under the receiver operating characteristic curve (AUC-ROC) values (determined with the pROC [1.18.0] package in R software) were used to evaluate model performance. Random forest model evaluation was performed using the entire validation cohort. For Kaplan–Meier survival curves, *P*-values were analyzed by two-tailed log-rank tests.

## Results

### Research plan

**Fig. 1:**
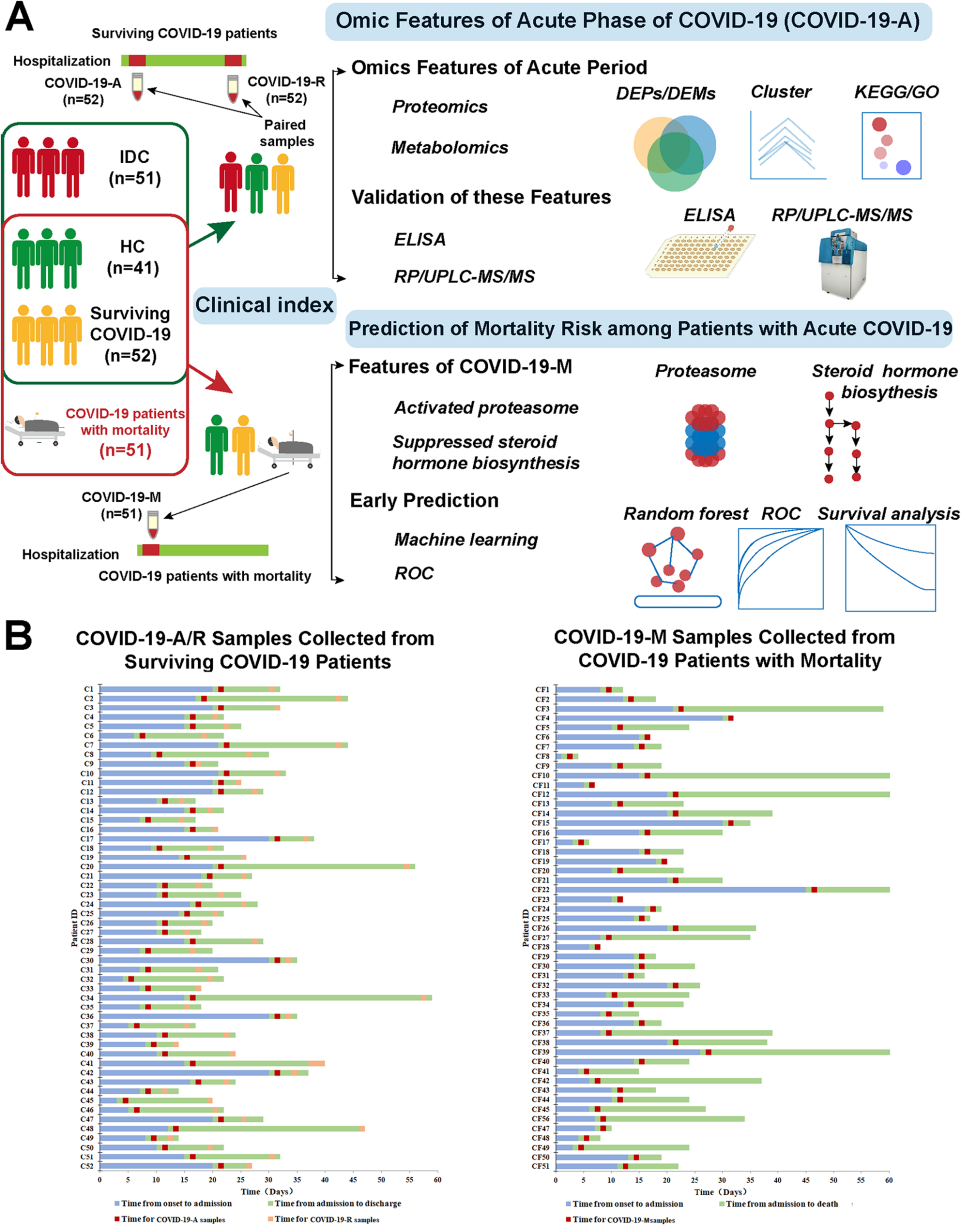
Study Overview. (**A**) Overview of assay modalities and validation methods. (**B**) Summary of COVID-19 COVID-19-A patients (*n* = 52) and COVID-19-M patients (*n* = 51). The y-axis displays patient identification numbers; the x-axis shows days since disease onset

Identification of acute phase characteristics can provide insights concerning key factors involved in the onset of acute COVID-19, with the potential to prevent disease progression. Thus far, few studies have investigated molecular changes in plasma samples between acute and recovered COVID-19 patients. Thus, we collected plasma samples from COVID-19 patients in the acute phase (COVID-19-A) and recovered phase (COVID-19-R), along with samples from IDCs and HCs, to analyze the molecular signatures of acute COVID-19 (Fig. 1A and Supplementary Data 1). Paired plasma samples were obtained from these COVID-19 patients within 1 day after hospital admission (COVID-19-A, red box, Fig. 1B) and 1–3 days before discharge (COVID-19-R, pink box, Fig. 1B). To ensure data reliability, typical molecular features were validated in an independent test cohort.

Next, we performed a comprehensive molecular analysis of COVID-19-M patients, with the goal of predicting mortality during the acute phase of disease (Fig. 1A). COVID-19-M samples were collected from COVID-19 patients with mortality at the next day of hospitalization, as red box represents the samples collected from this group of patients (Fig. 1B). Omics analyses indicated that, other than immunosuppression, impaired steroid hormone biosynthesis, and elevated inflammation, excessive proteasome activity was the most prominent signature in the acute phase of disease among patients with mortality. Based on these data, we developed a new biomarker panel using machine learning algorithms to predict COVID-19 mortality among surviving patients during the acute phase of disease. This plasma biomarker panel was then validated in a larger independent cohort.

### Clinical characteristics

We evaluated differences in basic clinical factors (including patient age, sex, and clinical indicators) among groups (Supplementary Data 2). In particular, we analyzed dysregulated clinical laboratory biomarkers in COVID-19 patients. Compared with HCs, IDCs and COVID-19 patients showed higher Neu counts. Conversely, there were decreases in the Lym and RBC counts, as well as the Hgb level, in IDCs and COVID-19 patients.

Compared with surviving COVID-19 patients, patients with mortality showed increased WBC and Neu counts, as well as a decreased Lym count. Moreover, the Lac level was higher, and the PaO_2_/FiO_2_ percentage was lower in patients with mortality than in surviving patients. Overall, the clinical indicator profiles indicated that breakthrough cases of COVID-19 were associated with the dysregulation of inflammation and immunity.

### Omics features of COVID-19 in patients with acute disease

#### Suppressed immunity and metabolism in the acute phase

**Fig. 2:**
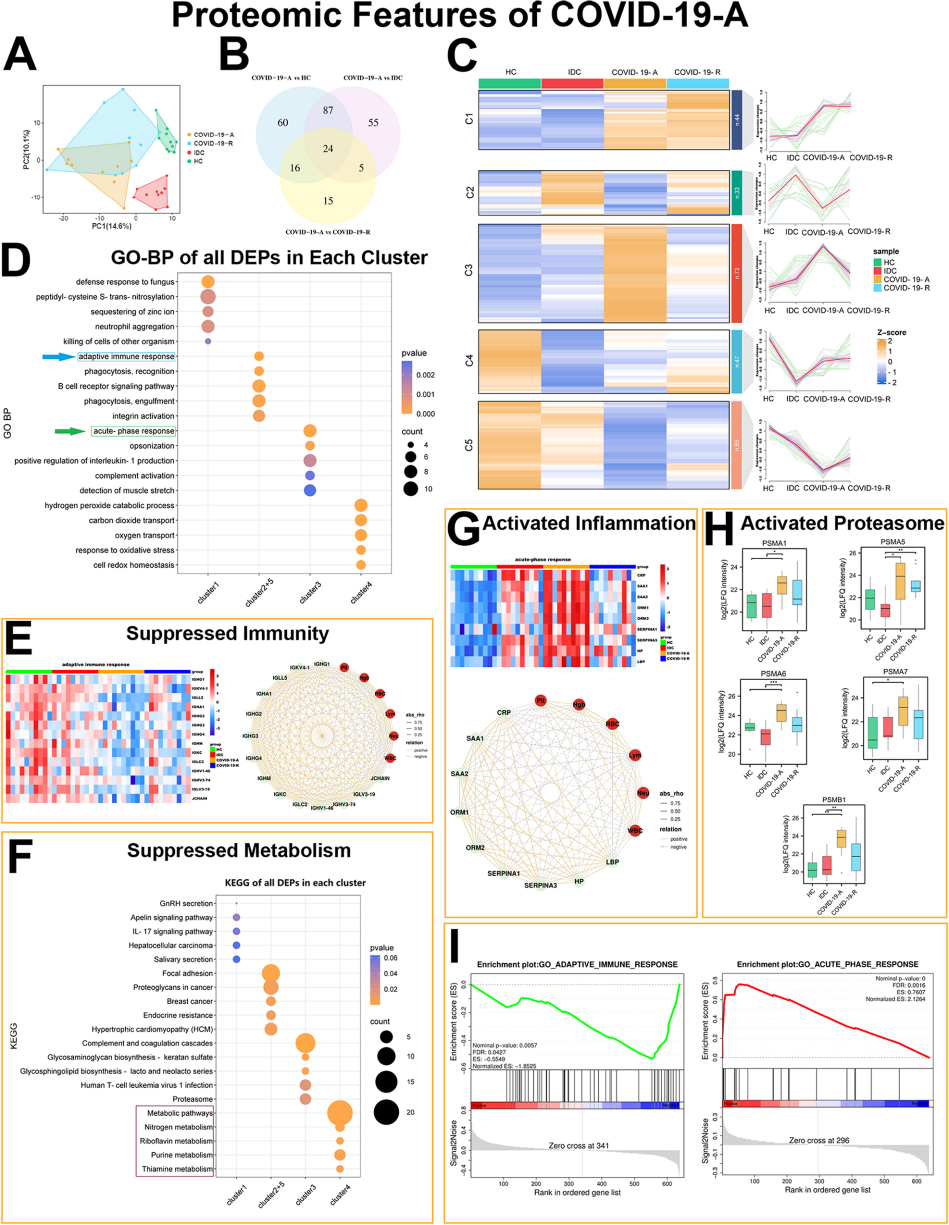
Plasma Proteome Analyses Reveal the Landscape of Host Responses in Patients with Acute COVID-19. (**A**) PLS-DA score plots for COVID-19-A, COVID-19-R, IDC, and HC groups. (**B**) Venn diagram of the numbers of DEPs among COVID-19-A, COVID-19-R, IDC, and HC groups. (**C**) Heatmap of 262 DEPs clustered using Mfuzz into five discrete significant clusters. (**D**) GO-BP enrichment analysis of all DEPs in each cluster, showing the top 5 GO terms. Green box highlights suppressed immunity in clusters 2 and 5. Blue box highlights enhanced inflammation in cluster 3. (**E**) Heatmap showing expression levels of DEPs related to suppressed immunity. Correlation analysis of immunity-associated proteins and clinical indexes. (**F**) KEGG terms for all DEPs in each cluster, showing the top 5 GO terms. Red box highlights metabolic suppression in cluster 4. (**G**) Heatmap showing expression levels of DEPs related to enhanced inflammation. Correlation analysis of inflammation-related proteins and clinical indexes. (**H**) Expression levels of altered proteasome subunits across the four groups. Statistical significance was determined by one-way ANOVA and Tukey’s HSD. *P < 0.05; **P < 0.01; ***P < 0.001. (**I**) GSEA to assess the enrichment of acute phase and adaptive immunity proteins during the acute phase of disease in COVID-19-A patients, compared with HCs

We examined the characteristics of COVID-19 in patients with acute disease, primarily focusing on the underlying mechanisms of host dysfunction after SARS-CoV-2 infection. PLS-DA was used to demonstrate separation among these groups (Fig. 2A). Our proteomic analysis showed that 262 proteins were differentially expressed in samples from COVID-19-A patients compared with controls (HCs, IDC and COVID-19-R samples, Fig. 2B and Supplementary Data 3.1). To characterize groupwise progressive changes in protein expression, we conducted unsupervised clustering of DEPs. This analysis revealed five distinct expression patterns across patients with different phases of disease, including an increasing cluster (1), two acute phase low clusters (2 and 5), an acute phase high cluster (3), and a “V” cluster (4) (Fig. 2C).

Intriguingly, DEPs in clusters 2 and 5 were significantly decreased in the COVID-19-A group compared with the COVID-19-R, HC, and IDC groups. GO analysis revealed that these proteins are related to the adaptive immune response, suggesting that the immune system is suppressed in the acute phase of COVID-19 (Fig. 2D). Key immunoglobulin proteins [Immunoglobulin heavy constant gamma (IGHG)1, IGHG2, IGHG3, IGHG4, Immunoglobulin lambda-like polypeptide (IGLL)5, Immunoglobulin heavy constant alpha (IGHA)1, Immunoglobulin heavy constant mu (IGHM), Immunoglobulin kappa constant (IGKC), Immunoglobulin lambda constant (IGLC)2, Immunoglobulin kappa variable (IGKV)4 − 1, Immunoglobulin heavy variable (IGHV)1–46, IGHV3-74, Immunoglobulin lambda variable (IGLV)3–19 and Immunoglobulin J chain (JCHAIN)] in the adaptive immune response shared this expression pattern (Fig. 2E and Fig. S1A). These protein expression patterns were positively correlated with the Lym, Neu, and WBC counts (Fig. 2E). Notably, KEGG analysis showed that proteins in cluster 4, which rapidly decreased before returning to normal levels, were associated with the suppressed metabolic function in the acute phase of COVID-19 (Fig. 2F). Moreover, the expression of these DEPs in COVID-19-A/COVID-19-R groups were higher than that in IDC group, implying that the expression of these DEPs might be more susceptible to other pathogens. Overall, these findings suggest that innate immunity proteins and metabolic proteins are highly suppressed in the acute phase of COVID-19, compared with the recovery phase and other control conditions.

### Enhanced inflammation and proteasomal activation in the acute phase

Next, we investigated proteins that were increased in the acute phase of COVID-19. Our results showed that DEPs in cluster 3 were enriched in the acute-phase response (Fig. 2D). The expression levels of these proteins were specifically increased in COVID-19-A patients compared with the other three groups. Important proteins in the acute-phase response, including CRP, SAA1, SAA2, ORM1, ORM2, Alpha-1-antitrypsin (SERPINA)1, Alpha-1-antichymotrypsin (SERPINA)3, haptoglobin (HP), and lipopolysaccharide-binding protein (LBP), showed this expression pattern (Fig. 2D). In addition to their increased expression levels in the COVID-19-A group, most of these proteins exhibited substantial decreases in the COVID-19-R group (Fig. 2G and Fig. S1B). Moreover, digital cytometry revealed lower WBC, Lym, and Neu counts in the acute phase of COVID-19. The levels of proteins in cluster 3 were negatively correlated with Lym and WBC counts. Thus, we concluded that proteins in cluster 3 reflect enhanced inflammation in the acute phase of COVID-19. Multiple proteasome subunits (e.g., PSMA1, PSMA5, PSMA6, PSMA7, and PSMB1) were upregulated in COVID-19 patients, especially during the acute phase of disease, potentially contributing to the dysregulation of proteasome activity (Fig. 2H). These results were supported by the GSEA findings (Fig. 2I).

### Suppressed steroid hormone biosynthesis in the acute phase

**Fig. 3:**
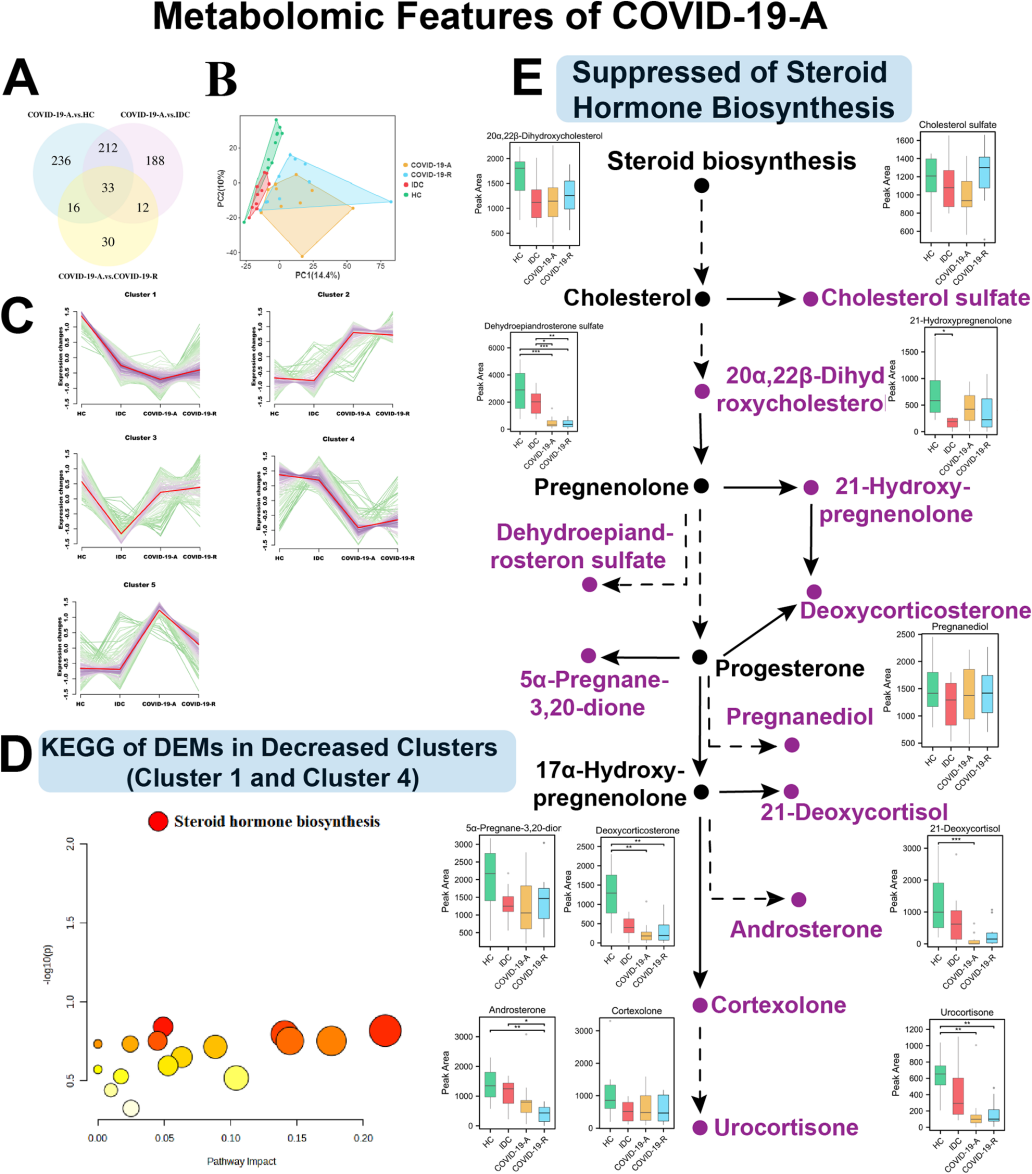
Plasma Metabolome Analyses Reveal Suppressed Steroid Hormone Biosynthesis in Patients with Acute COVID-19. (**A**) Venn diagram of DEMs among COVID-19-A, COVID-19-R, IDC, and HC groups. (**B**) PLS-DA score plots for COVID-19-A, COVID-19-R, IDC, and HC groups. (**C**) Cluster of DEMs. (**D**) KEGG terms enriched in clusters 1 and 4. (**E**) Many intermediates in the steroid hormone biosynthesis pathway were significantly decreased. Decreased metabolites are labeled in purple. Statistical significance was determined by one-way ANOVA and Tukey’s HSD. *P < 0.05; **P < 0.01; ***P < 0.001

Considering that metabolic inhibition may be a significant feature of the acute phase of COVID-19 (Fig. 2F), we performed metabolomic analyses, which revealed 2888 metabolites in the training cohort. Of these, 727 DEMs were significantly different in the COVID-19-A group, compared with the other three groups (Fig. 3A). PLS-DA was conducted to demonstrate separation among the groups (Fig. 3B). Moreover, we found that most DEMs in the COVID-19-A and COVID-19-R groups showed similar trends compared with HCs and IDCs (Fig. 3C); detailed data are provided in Supplementary Data 3.2.

We observed three distinct metabolite expression patterns across patients with different phases of disease, including two decreasing clusters (1 and 4), an increasing cluster (2 and 5), and a “V” cluster (3) (Fig. 3C). KEGG pathway analysis of DEMs from each cluster pattern indicated that the decreased expression in clusters 1 and 4 reflected a substantial impact of COVID-19 on steroid hormone biosynthesis (Fig. 3D). Steroid hormone metabolites have anti-inflammatory properties, which are important for the maintenance of immune homeostasis [23]. In the present study, several intermediates in the steroid hormone biosynthesis pathway (e.g., dehydroepiandrosterone sulfate, deoxycorticosterone, androsterone, 21-deoxycortisol, and urocortisone) were downregulated in COVID-19-A patients (Fig. 3E). Other metabolites, including 20α, 22β-dihydroxycholesterol, cholesterol sulfate, 21-hydroxypregnenolone, pregnanediol, 5α-pregnane-3,20-diol, and cortexolone, showed a decreasing trend in the COVID-19-A group compared with HCs. Additionally, compared with levels in COVID-19-A patients, the expression levels of many metabolites in COVID-19-R patients exhibited a slight shift toward levels observed in HCs. Overall, our findings suggest that steroid hormone metabolism is disrupted in the acute phase of COVID-19, which could contribute to COVID-19 pathogenesis by influencing host anti-inflammatory pathways.

### Validation of COVID-19-A features

**Fig. 4:**
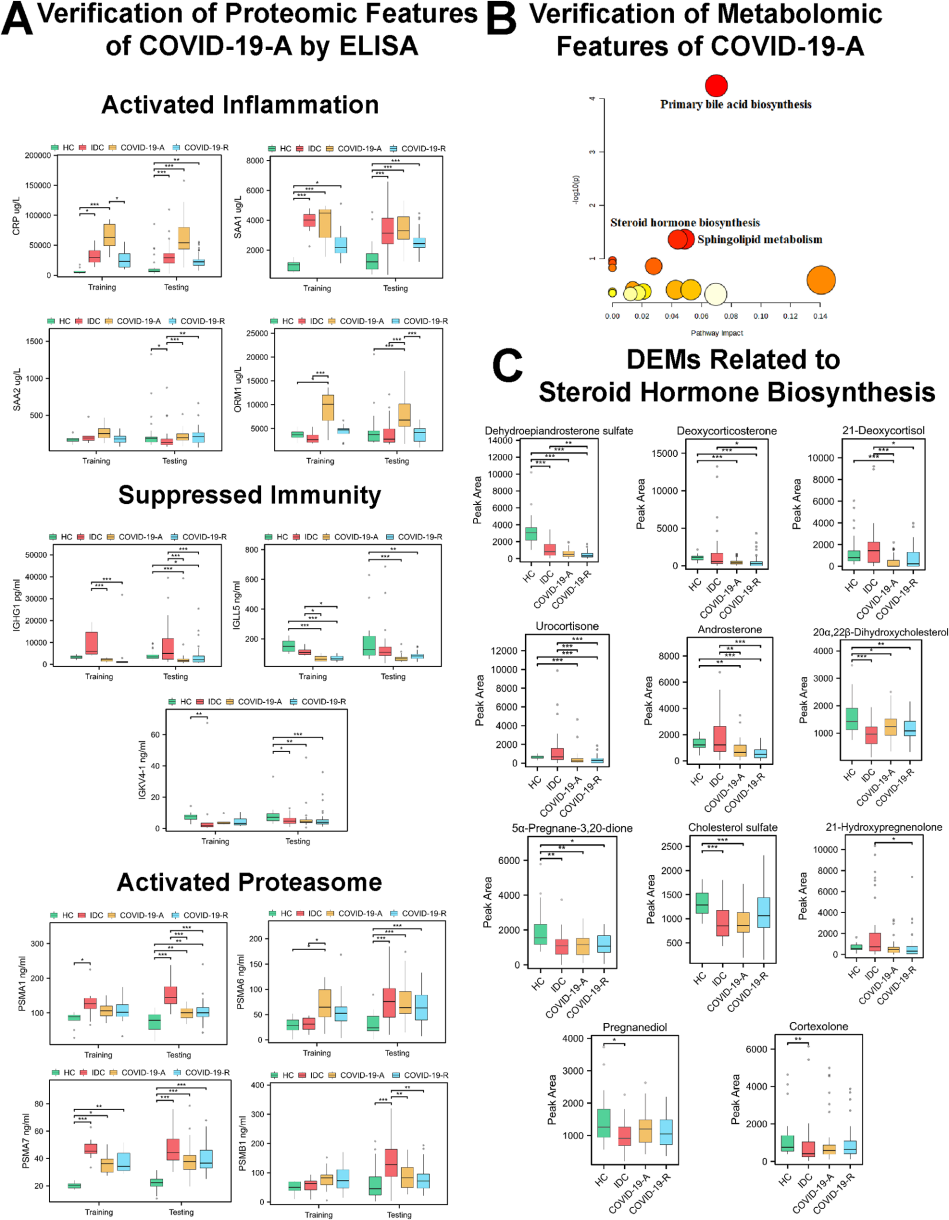
Validation of Typical Features Related to Acute COVID-19. (**A**) Validation of DEPs related to enhanced inflammation, suppressed immunity, and proteasomal activation by ELISA in the training and test cohorts, respectively. (**B**) KEGG terms for DEMs among patients in the test cohort. (**C**) Validation of DEMs related to steroid hormone biosynthesis. Statistical significance was determined by one-way ANOVA and Tukey’s HSD. *P < 0.05; **P < 0.01; ***P < 0.001

Thus far, our analyses revealed numerous changes in host plasma proteins and metabolites that may contribute to COVID-19 pathogenesis. To confirm the reliability of features identified in COVID-19-A patients, we conducted a larger-scale omics analysis of a validation cohort comprising COVID-19-A patients, COVID-19-R patients, HCs, and IDCs. We selected four inflammation-related proteins (CRP, SAA1, SAA2, and ORM1), three immunity-related proteins (IGHG1, IGLL5, and IGKV4-1), and four PSM proteins (PSMA1, PSMA6, PSMA7, and PSMB1) for ELISA-based validation in the training and test cohorts (Fig. 4A). These DEPs were chosen based on the following screening criteria: high fold change and association with the proteomic features of COVID-19-A discussed above.

We found that the expression levels of inflammation-related proteins (CRP, SAA1, SAA2, and ORM1) were substantially elevated in the acute phase of COVID-19, consistent with the proteomic results. The levels of immunity-related proteins (IGHG1, IGLL5, and IGKV4-1) were slightly decreased in the acute phase of COVID-19, confirming that immunity had been suppressed. Moreover, we observed that proteasome activity was enhanced in the acute phase of COVID-19 (Fig. 4A).

Similarly, we found that decreased DEMs in samples from the test cohort (31 HCs, 41 IDCs, and 42 COVID-19-A patients) were involved in three biological processes, including steroid hormone biosynthesis (Fig. 4B). Key metabolite changes are summarized in Fig. 4C. Metabolites including dehydroepiandrosterone sulfate, deoxycorticosterone, 21-deoxycortisol, urocortisone, androsterone, 20α, 22β-dihydroxycholesterol, 5α-pregnane-3,20-dione, and cholesterol sulfate were considerably decreased in the COVID-19-A group; they were slightly increased in the COVID-19-R group. Collectively, our results confirmed the reliability of the proteomic and metabolomic data; they also validated the involvement of these molecules in the pathogenesis of acute COVID-19.

### Prediction of mortality risk among patients with acute COVID-19

#### Proteomic features of patients with mortality—proteasomal activation

**Fig. 5:**
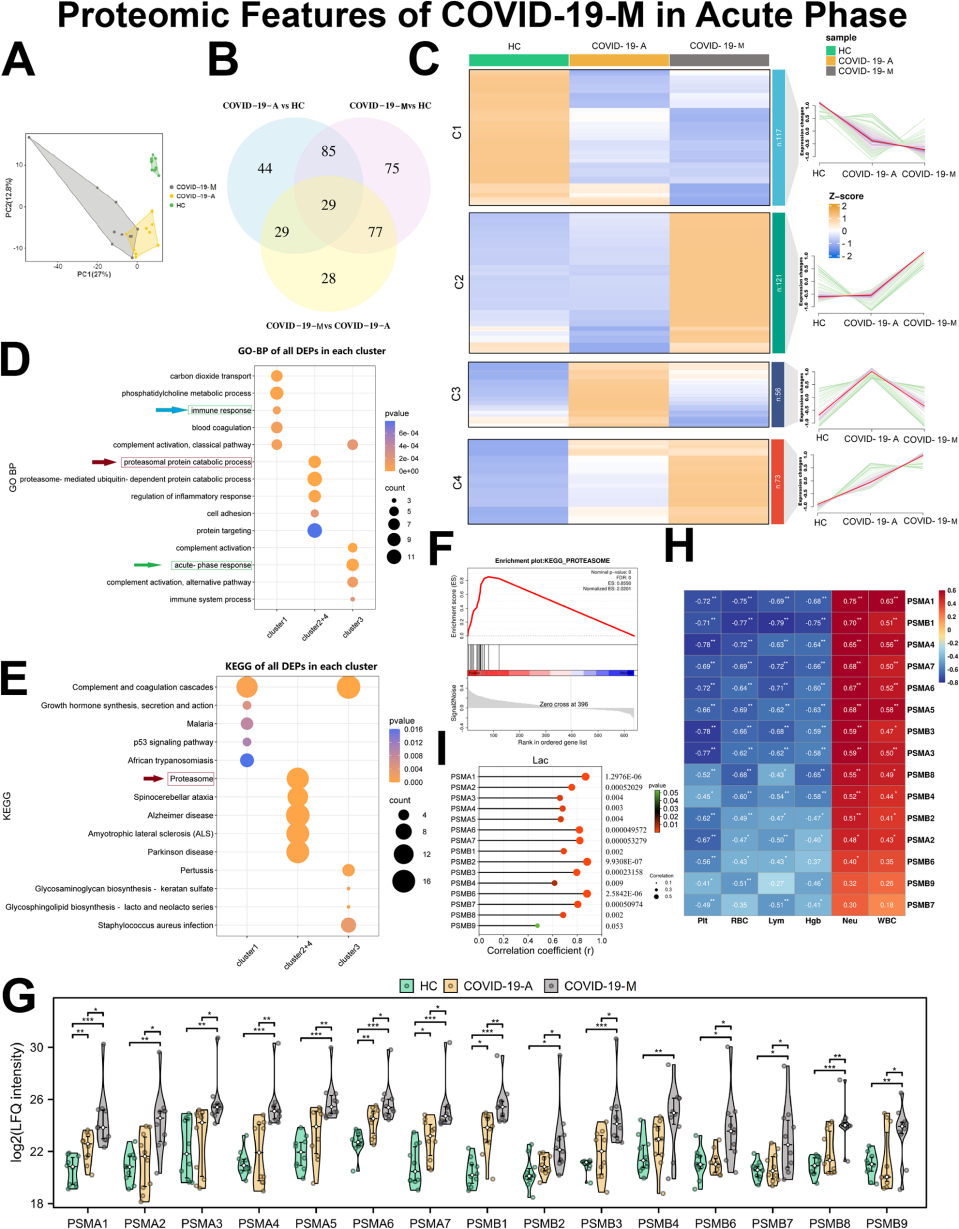
Proteomic Features of COVID-19-M Patients in the Acute Phase: Proteasomal Activation. (**A**) PLS-DA score plots for COVID-19-A, COVID-19-M, and HC groups. (**B**) Venn diagram of the number of DEPs among COVID-19-A, COVID-19-M, and HC groups. (**C**) Heatmap of 367 DEPs clustered using Mfuzz into four discrete significant clusters. (**D**) GO-BP enrichment analysis of all DEPs in cluster 1, clusters 2 and 4, and cluster 3, respectively. The top 5 GO terms are shown. (**E**) KEGG analysis of all DEPs in cluster 1, clusters 2 and 4, and cluster 3, respectively. The top 5 GO terms are shown. (**F**) Expression levels of proteasome subunits among COVID-19-A, COVID-19-M, and HC groups. Statistical significance was determined by one-way ANOVA and Tukey’s HSD. *P < 0.05; **P < 0.01; ***P < 0.001. (**G**) GSEA to assess the enrichment of proteasome signatures during the acute phase of disease in COVID-19-M patients, compared with COVID-19-A patients. ES, enrichment score; *P*-values were calculated via permutation test. (**H**) Correlation analysis of proteasome-associated proteins and clinical indexes. Red and blue numbers represent positive and negative correlations, respectively. (**I**) Correlation analysis of proteasome-associated proteins and Lac level in COVID-19 patients. *correlation *P* < 0.05. **correlation *P* < 0.01

In this study, we found that patients with acute COVID-19 could be clearly distinguished from HCs and IDCs using omic signatures. Next, we investigated the potential for these signatures to predict COVID-19 outcomes, using samples that had been collected from COVID-19 patients with mortality (COVID-19-M) during the acute phase of disease. In total, 946 proteins were quantified through a compound library search; 367 were differentially expressed among the COVID-19-M, COVID-19-A, and HC groups (Supplementary Data 3.3). PLS-DA (Fig. 5A) and a Venn diagram (Fig. 5B) were used to visualize the DEPs.

Furthermore, we observed three expression patterns across the comparison groups, including a decreasing cluster (1), two increasing clusters (2 and 4) and an inverted “V” cluster (3) (Fig. 5C). Consistent with proteomic features in the COVID-19-A group, suppressed immunity (Fig. 5D and Fig. S2A) and enhanced inflammation (Fig. 5D and Fig. S2B) were present in COVID-19-M patients.

Importantly, we observed that the abundances of proteasome subunits were increased in plasma from COVID-19-M patients. The ubiquitin − proteasome system is essential for protein degradation and thus closely associated with processes such as apoptosis, cell cycle regulation, and the inflammatory response [24]. Therefore, the proteasome serves as an intracellular indicator of health and disease. In this study, we found that DEPs in clusters 2 and 4, which showed rapidly increased expression in the COVID-19-M group, were mainly involved in the proteasomal protein catabolic process (Fig. 5D, GO-BP) and proteasome pathway (Fig. 5E, KEGG). As shown in Fig. 5F, nearly all proteasome subunits (e.g., PSMA1, PSMA2, PSMA3, PSMA4, PSMA5, PSMA6, PSMA7, PSMB1, PSMB2, PSMB3, PSMB6, PSMB7, PSMB8, and PSMB9) were considerably upregulated in COVID-19-M patients, compared with COVID-19 patients and HCs. These observations were supported by the enriched proteasome signature observed among COVID-19-M patients during the acute phase of disease (Fig. 5G). Moreover, we observed that the expression levels of proteasome subunit proteins were negatively associated with RBC and Plt counts (Fig. 5H); they were positively associated with the levels of Lac, a biomarker for disease severity. Lac is regarded as a danger signal that can affect the immune system [25, 26]; its association with proteasome subunits supports the hypothesis that proteasomal activation is involved in COVID-19 mortality (Fig. 5I). Thus, proteasome subunits, whose inhibitors reportedly are protective against SARS-CoV-2 infection, may be strong indicators of mortality risk in COVID-19 patients.

### Suppressed steroid hormone biosynthesis in patients with mortality

**Fig. 6:**
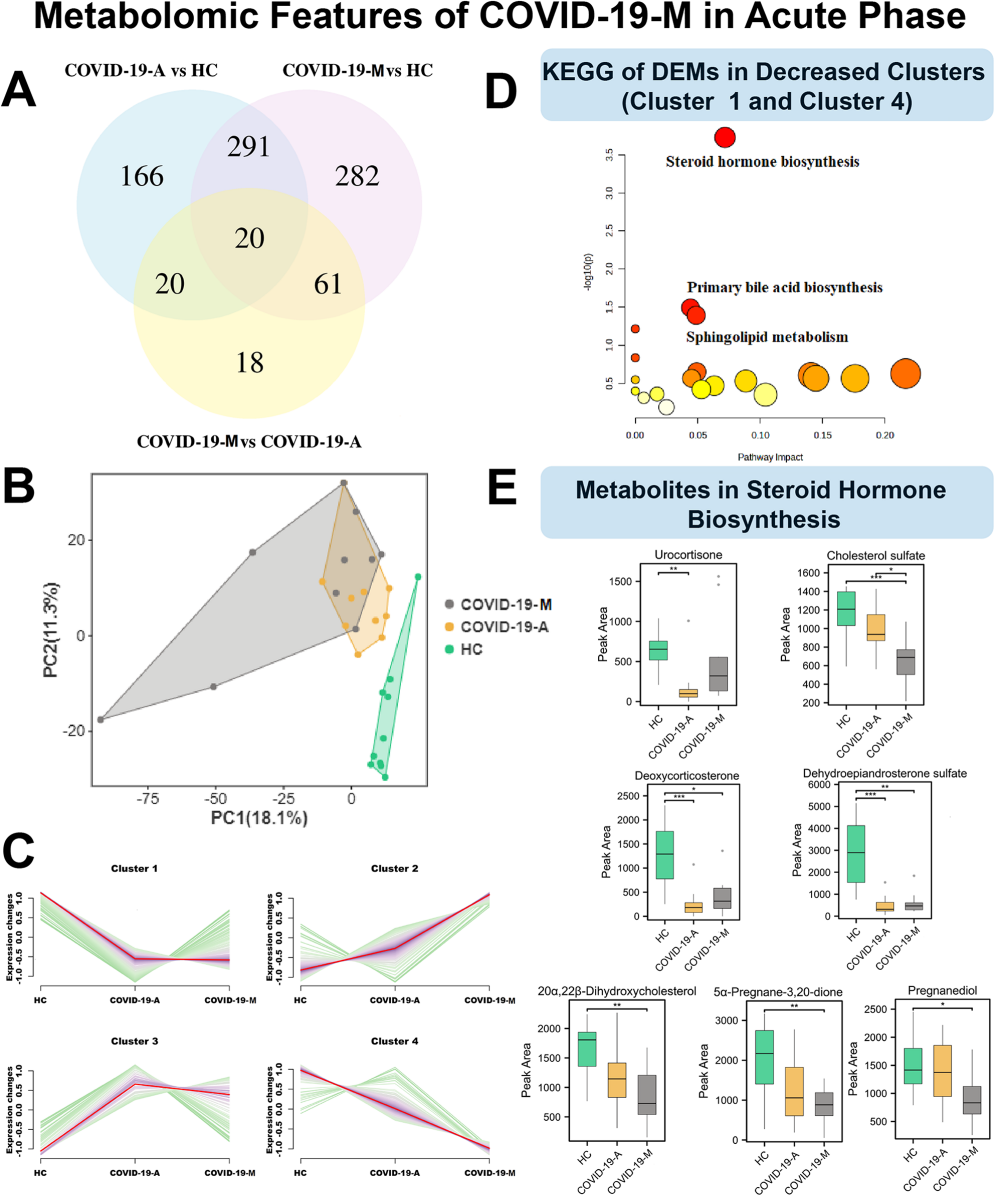
Metabolomic Features of COVID-19-M Patients in the Acute Phase: Suppressed Steroid Hormone Biosynthesis. (**A**) Venn diagram of the number of DEMs among COVID-19-A, COVID-19-M, and HC groups. (**B**) PLS-DA score plots for COVID-19-A, COVID-19-M, and HC groups. (**C**) Hierarchical clustering illustrating four DEP patterns across the three groups. (**D**) KEGG terms enriched in decreased clusters (1 and 4). (**E**) Expression of DEMs in the steroid hormone biosynthesis pathway. Statistical significance was determined by one-way ANOVA and Tukey’s HSD. *P < 0.05; **P < 0.01; ***P < 0.001

Considering the differences in plasma protein levels between survivors and non-survivors, we hypothesized that these differences could be more comprehensively visualized using the metabolome, which is widely regarded as the omics field that most closely resembles phenotyping. After data processing and annotation, we identified 3,345 metabolites, of which 858 were differentially expressed among the COVID-19-M, COVID-19-A, and HC groups (Supplementary 3.4). A Venn diagram (Fig. 6A) and PLS-DA (Fig. 6B) were used to visualize the separation among groups; clusters (Fig. 6C) were established to illustrate groupwise expression trends.

There was evidence of substantial steroid hormone biosynthesis suppression in COVID-19-M patients (Fig. 6D), although some downregulated metabolites differed from the metabolites identified in COVID-19-A patients. As shown in Fig. 6E, steroid hormone derivatives (e.g., urocortisone, cholesterol sulfate, deoxycorticosterone, dehydroepiandrosterone sulfate, 20α, 22β-dihydroxycholesterol, 5α-pregnane-3,20-dione, and pregnanediol) were downregulated in COVID-19-M patients. This downregulation probably resulted from macrophage modulation. Steroid hormones reportedly are able to promote macrophage activity, as well as the activities of other immune cells [27, 28] and non-immune cells [29]. The lack of steroid hormones, essential intermediates in corticosterone synthesis [30], may be the main cause of the host’s inability to defend against SARS-CoV-2 infection.

### Early prediction of poor prognosis based on features of COVID-19-M patients

**Fig. 7:**
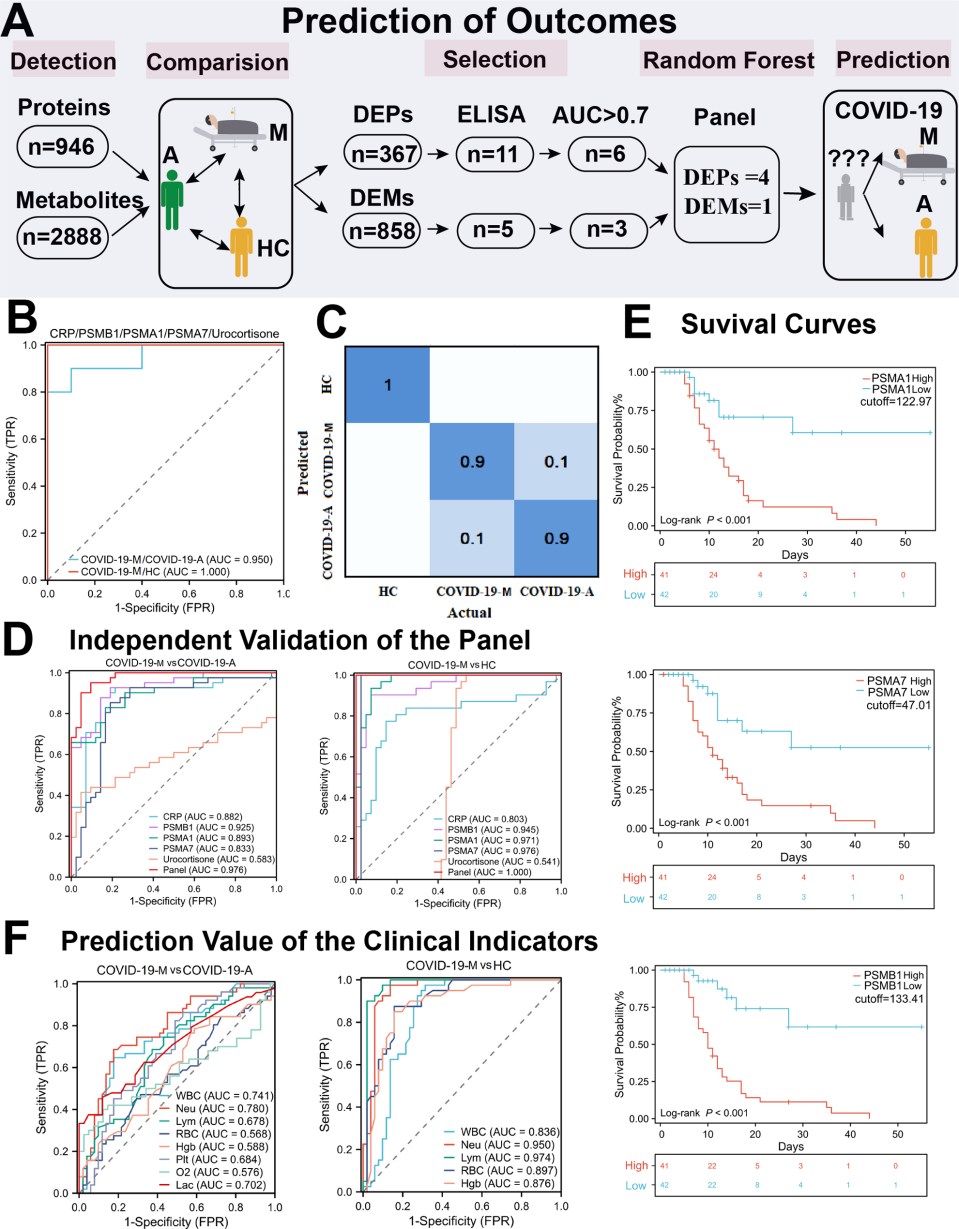
Identification and Validation of Potential Biomarkers for Prediction of Mortality Risk in COVID-19 Patients. (**A**) Workflow for predictive marker selection. (**B**) ROC curve illustrating the performance of classifiers based on the combination panel. The model was trained with 30 samples and evaluated by patient-based five-fold cross-validation. (**C**) Biomarker panel confusion matrix among different plasma samples. (**D**) AUC values for five biomarkers and the combined panel in distinguishing COVID-19-M patients from COVID-19-A patients and HCs in the validation cohort. The model was tested with 114 samples collected from COVID-19 patients and HCs, then evaluated by patient-based five-fold cross-validation. (**E**) Kaplan–Meier survival curves were established according to mortality risk score; optimal cutoff values were derived from X-tile (all *P* < 0.0001, log-rank test). Patients were divided into two groups based on the median expression levels of PSMA1, PSMA1, PSMA7, and PSMB1. *P*-values were calculated by two-tailed log-rank tests. (**F**) AUC values for clinical indexes in distinguishing COVID-19-M patients from COVID-19-A patients and HCs. The model was trained and tested using 144 samples collected from both training and test cohorts, then evaluated by patient-based five-fold cross-validation

Early prediction of mortality risk is important for efforts to identify and avoid possible causes of death. Considering that immunity, inflammation, proteasome activity, and steroid hormone biosynthesis were key features influencing the outcomes of COVID-19-M patients, we developed a new computational pipeline that used these features to predict poor COVID-19 prognosis (Fig. 7A). As shown in Fig. S3A, 11 typical DEPs (CRP, ORM1, SAA1, SAA2, IGHG1, IGKV4-1, IGLL5, PSMA1, PSMA6, PSMA7, and PSMB1) were validated by ELISA. As expected, there were significant differences in these DEPs; the observed ratios were consistent with the proteomic data (Fig. S3B, Supplementary Data 4. training cohort).

Based on the ELISA and metabolomic data, nine features including six DEPs and three DEMs with AUC > 0.7 were selected as candidates for prediction analysis. The AUC-ROC curves with optimal cutoffs for all features are shown in Supplementary Data 5 and Fig. S4. Next, three machine learning classifiers (logistic regression, random forest and linear support vector machine) were used to determine the optimal diagnostic model; accuracies and error rates were evaluated by 10-fold cross-validation. The diagnostic performance of these machine learning classifiers were expressed in Supplementary Data 6. Random forest classification identified four DEPs (CRP, PSMA1, PSMA7, and PSMB1) and one DEM (urocortisone) as the best diagnostic model combination. As presented in Fig. 7B, this panel had AUC values of 0.950 and 1.000 for distinguishing COVID-19-M patients from COVID-19-A patients and HCs, respectively. It was able to distinguish COVID-19-M patients from COVID-19-A patients with 90.0% sensitivity and 90.0% specificity (Fig. 7C). Notably, although some proteins in this model have been previously identified as potential biomarkers of COVID-19 [31, 32], this is the first study to link their expression levels with mortality.

### Independent validation

To estimate the predictive value of this new computational pipeline, we analyzed a randomized cohort constructed according to ELISA and metabolomic data (Fig. S3C). The diagnostic performance of the three machine learning classifiers were expressed in Supplemental Data 6. We found that the levels of PSMA1, PSMA7, PSMB1, and urocortisone were considerably higher in plasma from COVID-19-M patients than in plasma from COVID-19-A patients or HCs (Fig. S3B, Supplementary Data 4. testing cohort). Additionally, the levels of steroid hormone biosynthesis metabolites were lower in plasma from COVID-19 patients than in plasma from HCs (Fig. S3D, test cohort). As shown in Fig. 7D, the AUC values of this panel for distinguishing COVID-19-M patients from COVID-19-A patients and HCs were 0.976 and 1.000, respectively. Comparisons of each protein/metabolite individually or in combination showed that the individual DEPs and DEMs were effective in terms of distinguishing COVID-19-M patients from COVID-19-A patients (Fig. 7D). Kaplan–Meier analysis indicated that increased expression of the proteasome cluster was correlated with mortality among COVID-19 patients, confirming the value of these proteins in terms of predicting poor prognosis (Fig. 7E).

Finally, we evaluated the predictive value of classical disease severity indicators in comparison with our novel pipeline. As shown in Fig. 7F, AUC values were 0.741 for WBC count, 0.780 for Neu count, and 0.702 for Lac level in distinguishing COVID-19-M patients from COVID-19-A patients. Other classical clinical indicators including Lym count, RBC count, Hgb level, and Plt count had considerably lower AUC values (0.568–0.684) for the prediction of poor prognosis. Furthermore, O_2_ saturation levels reportedly can explain clinical deterioration and mortality in COVID-19 patients [33]. In this study, the AUC value of PaO_2_/FiO_2_ for predicting poor prognosis was 0.576, which was substantially lower than the AUC value for the novel pipeline (0.976). Collectively, our results confirmed the reliability of the multi-omics data. They also demonstrated that the model constructed in this study has great potential for predicting mortality risk in COVID-19 patients at the time of hospitalization, such that it outperformed clinical metrics.

## Discussion

Highly contagious SARS-CoV-2 variants continue to strain health systems worldwide. Although most affected individuals are asymptomatic or have mild disease, some individuals develop severe disease with the potential for rapid death. Thus far, most studies have focused on plasma molecular signatures related to COVID-19 severity [16, 20, 32, 34]. Few studies have conducted multi-dimensional analysis of the host response to SARS-CoV-2 infection during the acute and recovery phases. Moreover, the potential for rapid disease progression underscores the need for methods that can reliably predict survival among hospitalized patients. Accordingly, we conducted untargeted MS/MS-based proteomics and metabolomics to evaluate the features of patients with acute COVID-19. Additionally, the collection of early clinical samples at the time of hospitalization allowed exploration of mortality risk in such patients. Our analysis of plasma samples showed that most COVID-19 patients could be clearly distinguish from HCs, regardless of the time point or outcome. Through this omics analysis, we confirmed the findings of dysregulated inflammation, immunity, proteasome activity, and steroid hormone biosynthesis. We also identified a predictive panel that could be utilized at hospitalization to assess COVID-19 mortality.

A key finding in the present study was the link between suppressed immunity and death, despite the more pronounced suppression observed in samples from patients with acute COVID-19; this finding suggests that the host response is impaired in patients with mortality. Notably, multiple immunoglobulin chains (e.g., IGHG1, IGKV4-1, IGHG3, IGHV1-46, IGHA1, IGHG2, IGLC2, IGKV2-29, and IGKC) exhibited substantially lower expression in COVID-19-A patients; they showed slightly higher expression in COVID-19-R patients. These changes in the levels of immunity-related proteins were negatively correlated with Lym, Neu, and WBC counts; changes in the levels of other immunity-related proteins were also associated with these clinical indicators.

Metabolomics data analysis also revealed that the steroid hormone synthesis pathway is significantly inhibited in COVID-19 patients. Up to now, a large number of metabolomics studies have been carried out in COVID-19, and researchers have identified a variety of COVID-19-related metabolites, including multiple pathways such as glucose metabolism, urea cycle, and lipid metabolism [35, 36]. Ding Shi et al. verified the predictive ability of the combination of 7 metabolites on the severity of COVID-19 disease, including steroid substances, which was similar to our research results [37]. It should be emphasized that previous studies focused on determining the severity of a patient’s disease through metabolic markers. In this study, we mainly focus on the typical DEPs in the acute phase of the disease, which be able to predict the poor prognosis. Besides, it has been reported the close link between cytokine disorders in COVID-19 patients and certain metabolites such as choline and alpha-ketoglutaric acid, strongly suggesting potential therapeutic targets [34]. Kaiming Wang et al. also revealed persistent inflammatory responses, platelet degranulation, and cell activation in multiple dysregulated metabolic pathways with in long COVID-19 patients [38].

Furthermore, our proteomic and metabolomic data enabled systematic analysis of the molecular pathogenesis of COVID-19 in patients with mortality. We observed increases in many plasma proteins (e.g., CRP) during the acute phase, consistent with previously report [16]. These increases could lead to enhanced cytokine and chemokine secretion, possibly triggering a cytokine storm; they also can cause excessive recruitment of macrophages from peripheral blood, contributing to acute injury [39, 40]. Moreover, the expression levels of SAA1, SAA2, ORM1, ORM2, SERPINA1, SERPINA3, LBP, and HP were substantially elevated in samples from COVID-19-A patients. Many of these proteins, such as SERPINA3, ORM1 and ORM2, have been used to distinguish between mild and severe cases of COVID-19 [32]. Intriguingly, the activation of inflammation was restored to a certain extent in COVID-19 patients with mortality than surviving patients at the time of hospitalization. This result is likely related to the poor physical responsiveness of the non-surviving patients, although their clinical manifestations were similar to the manifestations of surviving patients.

The most important findings of the present study were the striking changes in proteasome subunit levels between surviving and non-surviving COVID-19 patients. Although there were no significant differences in clinical presentation or laboratory examination among surviving patients at the time of hospitalization, the plasma levels of proteasome subunits substantially differed. Our results suggest that proteasome subunits can aid in predicting the outcomes of COVID-19 patients. Indeed, the proteasome has been described as the basis of several diseases; it has also been identified as an early prognostic biomarker for sepsis, primarily in association with lymphocyte apoptosis [41]. Moreover, the main roles of the proteasome are recognition, binding, and degradation of ubiquitinated proteins. The ubiquitin–proteasome system is closely involved in regulating the antiviral immune response [42]. In this regard, some proteasome inhibitors have shown efficacy in limiting the life cycles of viruses such as SARS-CoV-2 [42, 43]. Previous studies indicated that proteasome inhibitors can induce apoptosis [44, 45]; this property underlies their frequent application in cancer treatment [27, 46]. In the context of COVID-19, Longhitano et al. demonstrated that proteasome inhibitors had therapeutic effects [42, 47]. Xue et al. also observed the upregulation of some proteasome subunits, although their associations with hypoxemia and hyperinflammation require clarification [31]. We hypothesize that viral infection drives local and systemic hyperinflammatory responses, leading to dysregulated proteasome activity that could be involved in COVID-19 pathogenesis. Further research is needed to determine whether and how proteasome subunit overexpression contributes to COVID-19 mortality.

Another critical observation was the suppression of steroid hormone biosynthesis in COVID-19 patients, especially patients in the COVID-19-M group. There is evidence that steroid hormones play essential roles in the adaptive immune response and are involved in regulatory processes during infection [48]. Moreover, steroid hormones (e.g., progesterone, androgens, and estrogens) reportedly can enhance the activities of many immune cells and non-immune cells [23]. We observed reduced expression of 21-hydroxypregnenolone, an important intermediate during corticosterone synthesis, implying that corticosterone biosynthesis is suppressed in patients with SARS-CoV-2 infection. Our results suggest that appropriate corticosteroid supplementation could maintain hormonal balance, thereby modulating the inflammatory response and reducing mortality risk.

After the identification of significant DEPs and DEMs between surviving and non-surviving COVID-19 patients, we utilized machine learning to detect robust features that are predictive of COVID-19 mortality, with the potential to determine poor prognosis at the time of hospitalization. Although separate proteomic and metabolomic biomarkers exhibited AUC values > 0.7 for predicting adverse outcomes, the combination of four proteins (CRP, PSMA1, PSMA7, and PSMB1) and one metabolite (urocortisone) achieved an AUC of 0.976. To test the predictive power of this survival model, we used it to analyze a test cohort comprising 83 COVID-19 patients and 31 HCs. The results showed that our panel exhibited > 90% accuracy for predicting poor prognosis in the test cohort. Furthermore, the diagnostic efficacy of this combined biomarker is significantly better than the efficacies of other clinical indicators (WBC, Neu, RBC, Lym, and Plt counts; Hgb and Lac levels; and oxygenation index). Thus, our combined biomarker could effectively predict poor COVID-19 prognosis.

Our findings have several potential clinical benefits. First, larger cohorts are needed to validate biomarker panels that can predict mortality among COVID-19 patients at the time of hospitalization, facilitating early intervention. Such predictions may allow patients with acute COVID-19 to receive more effective preventive treatments. Our combined biomarker could also serve as a useful indicator of the therapeutic effects of potential treatments for COVID-19. Finally, our results suggest that specific host responses contribute to the heterogeneous outcomes of COVID-19.

## Conclusion

This study is informative in elucidating the trajectories of COVID-19, with pointing to dysregulation of inflammation, immunity, proteasome activity, and steroid hormone biosynthesis. And machine learning panel is constructed to predict mortality among COVID-19 patients on the first day of hospitalization, providing clues for new interventions. More clinical evidence is still awaited to support the findings here.

### Electronic supplementary material

Below is the link to the electronic supplementary material.

Supplementary Material 1

Supplementary Material 2

Supplementary Material 3

Supplementary Material 4

Supplementary Material 5

Supplementary Material 6

Supplementary Material 7

## Data Availability

Correspondence and requests for data should be addressed to Prof. Jieqiong Li, Prof. Nan Song and Prof. Zhaohui Tong.

## Abbreviations

COVID-19: Coronavirus disease 2019
IDCs: Other infectious diseases
HCs: Healthy controls
ELISA: Enzyme-linked immunosorbent assays
SARS-CoV-2: Severe acute respiratory syndrome coronavirus 2
COVID-19-A: Acute COVID-19
COVID-19-R: Recovered COVID-19
COVID-19-M: COVID-19 patients with mortality
RT-PCR: Reverse transcription polymerase chain reaction
WBC: White blood cell
Neu: Neutrophil
Lym: Lymphocyte
RBC: Red blood cell
Hgb: Hemoglobin
Plt: Platelet
Lac: Lactic acid
PaO_2_/FiO_2_: Oxygenation index
LC-MS/MS: Liquid chromatography-tandem mass spectrometry
CRP: C-reactive protein
SAA1: Serum amyloid A-1
ORM1: Alpha-1-acid glycoprotein 1
IGHG1: Immunoglobulin heavy constant gamma 1
IGLL5: Immunoglobulin lambda-like 5
PSMA1: Proteasome subunit alpha type-1
PSMB1: Proteasome subunit beta type-1
MeOH: Methanol
ACH: Acetylcholine
UPLC-MS/MS: Ultra performace liquid chrmatography-electrospray tandem mass spectrometry
DEPs: Differentially expressed proteins
DEMs: Differentially expressed metabolites
ANOVA: One-way analysis of variance
HSD: Tukey’s honestly significant difference
PLS-DA: Partial least squares discriminant analysis
GO: Gene Ontology
KEGG: Kyoto Encyclopedia of Genes and Genomes
GSEA: Gene set enrichment analysis
AUC-ROC: Area under the receiver operating characteristic curve
IGHG1: Immunoglobulin heavy constant gamma 1
IGLL5: Immunoglobulin lambda-like polypeptide 5
IGHA1: Immunoglobulin heavy constant alpha 1
IGHM: Immunoglobulin heavy constant mu
IGKC: Immunoglobulin kappa constant
IGLC2: Immunoglobulin lambda constant 2
IGKV4-1: Immunoglobulin kappa variable 4 − 1
IGHV1-46: Immunoglobulin heavy variable 1–46
IGLV3-19: Immunoglobulin lambda variable 3–19
JCHAIN: Immunoglobulin J chain
SERPINA 1: Alpha-1-antitrypsin 1
SERPINA 3: Alpha-1-antichymotrypsin 3
HP: Haptoglobin
LBP: Lipopolysaccharide-binding protein
