# Supplementary material for: Dysregulated proteasome activity and steroid hormone biosynthesis are associated with mortality among patients with acute COVID-19
Source: J Transl Med. 2024 Jul 4;22:626. doi: 10.1186/s12967-024-05342-0 (PMC11229496; doi:10.1186/s12967-024-05342-0)
Supplement: Supplementary file 2 — Supplementary Material 2 [file 12967_2024_5342_MOESM2_ESM.docx]

**Supplementary Data 2: The clinical information and conducted biochemical laboratory tests.**

| Characteristics | IDC  (n=51) | HC  (n=41) | Surviving COVID-19  (n=52) | COVID-19 patients with mortality  (n=51) | P value a | P value b |
| --- | --- | --- | --- | --- | --- | --- |
| Gender (male/female) | 37/14 | 28/13 | 34/18 | 33/18 | 0.733 | 0.931 |
| Age (years) | 63.00(52.00-70.00) | 73.00(55.00-78.00) | 75.00(65.00-83.75) ^cd^ | 77.00(69.00-86.00) ^g^ | 0.345 | 0.743 |
| Days of hospitalization | 14.00(7.75- 24.25) | / | 11.50(7.25-14.75) | 10.00(4.00-18.00) | 0.137 | 0.121 |
| Critical score ^h^ | | | | | | |
| WBC, 10^9^/L | 7.67(4.66-15.86) ^e^ | 6.19(5.07-7.26) | 7.18(4.64-8.63) | 9.99(7.76-13.67) ^fg^ | 0.047 | <0.001 |
| Neu, 10^9^/L | 5.78(3.16-13.39) ^e^ | 3.59 (2.52-4.09) | 5.09(3.21-7.61) ^d^ | 8.93(6.08-11.89) ^fg^ | <0.001 | <0.001 |
| Lym, 10^9^/L | 0.99(0.48-1.55) ^e^ | 2.11(1.65-2.58) | 0.89(0.43-1.31) ^d^ | 0.48(0.36-0.75) ^fg^ | <0.001 | <0.001 |
| RBC, 10^12^/L | 3.97(3.31-4.37) ^e^ | 5.01(4.50-5.22) | 4.01(3.63-4.41) ^d^ | 3.77(3.29-4.19) ^g^ | <0.001 | <0.001 |
| Hgb, g/L | 120.50(100.00-130.00) ^e^ | 152.50(135.50-158.25) | 124.00(111.00-135.00) ^d^ | 117.00(105.00-126.00) ^g^ | <0.001 | <0.001 |
| Plt, 10^9^/L | 223.00(166.75-331.75) | 257.00(237.00-299.25) | 218.00(151.00-284.00) ^d^ | 151.00(103.00-213.00) ^fg^ | 0.027 | <0.001 |
| Lac, mmo/L | / | / | 1.5(1.15-2.1) | 2.10(1.58-3.73) | / | <0.001 |
| PaO_2_/FiO_2_ | 279.31(165.43-348.60) | / | 272.41(224.14-304.48) | 149.52(99.31-217.24) | 0.890 | <0.001 |

^a^ P value among IDC, HC and surviving COVID-19;

^b^ P value among HC, surviving COVID-19 and COVID-19 patients with mortality;

^c^ P value between Surviving COVID-19 and IDC;

^d^ P value between Surviving COVID-19 and HC;

^e^ P value between Surviving IDC and HC;

^f^ P value between COVID-19 patients with mortality and Surviving COVID-19;

^g^ P value between COVID-19 patients with mortality and HC;

^h^ Data are presented as median (percentiles);

COVID-19-A/R, surviving COVID-19 patients; COVID-19-M, COVID-19 patients with mortality; IDC,other infectious disease control; HC, healthy control; WBC, white blood cell; Neu, neutrophil; Lym, lymphocyte; RBC, red blood cell; Hgb: hemoglobin; Plt: platelet; Lac: lactic acid; PaO_2_/FiO_2_: oxygenation index.
