# Supplementary material for: Dysregulated proteasome activity and steroid hormone biosynthesis are associated with mortality among patients with acute COVID-19
Source: J Transl Med. 2024 Jul 4;22:626. doi: 10.1186/s12967-024-05342-0 (PMC11229496; doi:10.1186/s12967-024-05342-0)
Supplement: Supplementary file 4 — Supplementary Material 4 [file 12967_2024_5342_MOESM4_ESM.docx]

**Supplementary Data 4: The clinical information and conducted biochemical laboratory tests**

| Characteristics | Training Cohort (n=30) | | |  | Testing Cohort (n=114) | | | P value a | P value b | P value c |
| --- | --- | --- | --- | --- | --- | --- | --- | --- | --- | --- |
|  | COVID-19-A  (n=10) | COVID-19-M  (n=10) | HC  (n=10) |  | COVID-19-A  (n=42) | COVID-19-M  (n=41) | HC  (n=31) |  |  |  |
| Gender (male/female) | 6/4 | 5/5 | 5/5 |  | 28/14 | 28/13 | 23/8 | 0.723 | 0.212 | 0.224 |
| Age (years) | 60.00  (54.50-64.25) | 69.50  (57.25-77.50) | 64.30  (56.00-69.00) |  | 78.00  (70.00-85.00) | 81.00  (72.00-87.00) | 71.24  (66.00-72.00) | <0.001 | 0.003 | 0.045 |
| Days of hospitalization | 13.00  (11.25-16.75) | 11.50  (3.50-26.75) | / |  | 10.00  (7.00-14.50) | 9.00  (4.00-15.50) | / | 0.089 | 0.643 | / |
| Critical score d | | | | | | | | | | |
| WBC, 10^9^/L | 5.69  (3.74-7.86) | 12.36  (8.82-16.78) | 6.16  (5.45-7.38) |  | 7.35  (4.88-9.02) | 9.73  (7.34-13.31) | 6.21  (5.04-7.32) | 0.207 | 0.081 | 0.531 |
| Neu, 10^9^/L | 3.85  (3.15-7.19) | 11.21  (8.32-15.84) | 3.57  (2.96-3.76) |  | 5.59  (3.34-7.65) | 8.59  (6.01-11.56) | 3.60  (2.44-4.19) | 0.335 | 0.021 | 0.893 |
| Lym, 10^9^/L | 0.58  (0.35-1.39) | 0.36  (0.28-0.46) | 1.85  (1.60-2.65) |  | 0.90  (0.47-1.32) | 0.56  (0.37-0.82) | 2.13  (1.68-2.56) | 0.458 | 0.082 | 0.925 |
| RBC, 10^12^/L | 4.30  (3.76-4.51) | 2.53  (2.32-3.65) | 4.86  (4.32-5.07) |  | 3.97  (3.42-4.36) | 3.92  (3.62-4.28) | 5.10  (4.76-5.24) | 0.190 | <0.001 | 0.196 |
| Hgb, g/L | 132.00  (113.50-135.50) | 83.50  (72.25-106.25) | 152.00  (134.00,157.50) |  | 123.50  (110.75-135.25) | 119.00  (112.00-128.00) | 153.00  (137.50-159.00) | 0.474 | <0.001 | 0.813 |
| Plt, 10^9^/L | 262.00  (146.50-344.50) | 84.50  (44.00-111.25) | 251.00  (222.00-278.50) |  | 207.50  (150.25-271.00) | 165.00  (129.00-215.00) | 263.00  (238.00-311.00) | 0.428 | 0.003 | 0.572 |
| Lac, mmol/L | 1.4  (1.15-1.58) | 2.05  (1.60-3.50) |  |  | 1.6  (1.15-2.25) | 2.10  (1.58-3.83) |  | 0.214 | 0.921 |  |
| PaO_2_/FiO_2_ | 258.62  (224.14-341.28) | 99.42  (73.77-291.76) |  |  | 227.42  (217.58-303.62) | 166.94  (109.33-217.24) |  | 0.872 | 0.293 |  |

^a^ P value between COVID-19-A in training cohort and testing cohort;

^b^ P value between COVID-19-M in training cohort and testing cohort COVID-19-M and HC;

^c^ P value between HC in training cohort and testing cohort;

^d^ Data are presented as median (percentiles).

COVID-19-A, COVID-19 patients in acute phase; COVID-19-M, COVID-19 patients with mortality; HC, healthy control; WBC, white blood cell; Neu, neutrophil; Lym, lymphocyte; RBC, red blood cell; Hgb: hemoglobin; Plt: platelet; Lac: lactic acid; PaO_2_/FiO_2_: oxygenation index.
