# Supplementary material for: Dysregulated proteasome activity and steroid hormone biosynthesis are associated with mortality among patients with acute COVID-19
Source: J Transl Med. 2024 Jul 4;22:626. doi: 10.1186/s12967-024-05342-0 (PMC11229496; doi:10.1186/s12967-024-05342-0)
Supplement: Supplementary file 5 — Supplementary Material 5 [file 12967_2024_5342_MOESM5_ESM.doc]

**Supplementary Data 5. The AUC of the ROC curves calculated at optimal cutoffs**

| Features | Type | COVID-19-M vs HC | | COVID-19-M vs COVID-19-A | |
| --- | --- | --- | --- | --- | --- |
|  |  | AUC | CI | AUC | CI |
| CRP | DEP | 0.96 | 0.89 - 1.00 | 0.81 | 0.61 - 1.00 |
| ORM1 | DEP | 0.55 | 0.27 - 0.83 | 0.88 | 0.72 - 1.00 |
| SAA1 | DEP | 1.00 | 1.00 - 1.00 | 0.48 | 0.20 - 0.76 |
| SAA2 | DEP | 0.62 | 0.36 - 0.88 | 0.82 | 0.61 - 1.00 |
| IGHG1 | DEP | 0.98 | 0.93 - 1.00 | 1.00 | 1.00 - 1.00 |
| IGKV4-1 | DEP | 0.52 | 0.23 - 0.81 | 0.7 | 0.43 - 0.97 |
| IGLL5 | DEP | 0.58 | 0.31 - 0.85 | 0.92 | 0.80 - 1.00 |
| PSMA1 | DEP | 0.99 | 0.96 - 1.00 | 0.86 | 0.69 - 1.00 |
| PSMA6 | DEP | 1.00 | 1.00 - 1.00 | 0.71 | 0.46 - 0.96 |
| PSMA7 | DEP | 1.00 | 1.00 - 1.00 | 0.88 | 0.73 - 1.00 |
| PSMB1 | DEP | 1.00 | 1.00 - 1.00 | 0.96 | 0.89 - 1.00 |
| 20a,22b-Dihydroxycholesterol | DEM | 0.88 | 0.73 - 1.00 | 0.70 | 0.45 - 0.94 |
| Cholesterol sulfate | DEM | 0.91 | 0.77 - 1.00 | 0.79 | 0.57 - 1.00 |
| Pregnanediol | DEM | 0.82 | 0.63 - 1.00 | 0.73 | 0.49 - 0.96 |
| 5a-Pregnane-3,20-dione | DEM | 0.85 | 0.66 - 1.00 | 0.60 | 0.32 - 0.87 |
| Urocortisone | DEM | 0.70 | 0.44 - 0.96 | 0.78 | 0.56 - 0.99 |
| Dehydroepiandrosterone sulfate | DEM | 0.96 | 0.89 - 1.00 | 0.60 | 0.33 - 0.86 |

COVID-19-A, samples collected from acute phase of surviving COVID-19 patients; COVID-19-M, samples collected from COVID-19 patients with mortality; DEP, differentially expressed protein; DEM, differentially expressed metabolite; AUC, area under curve; CI, confidence interval; ROC, receiver operating characteristic.
