# Supplementary material for: Dysregulated proteasome activity and steroid hormone biosynthesis are associated with mortality among patients with acute COVID-19
Source: J Transl Med. 2024 Jul 4;22:626. doi: 10.1186/s12967-024-05342-0 (PMC11229496; doi:10.1186/s12967-024-05342-0)
Supplement: Supplementary file 6 — Supplementary Material 6 [file 12967_2024_5342_MOESM6_ESM.doc]

**Supplementary Data 6.The diagnostic performance of these machine learning classifiers**

| Machine Learning | group | Training cohort (n=30) | | | | Testing cohort (n=114) | | | |
| --- | --- | --- | --- | --- | --- | --- | --- | --- | --- |
|  |  | AUC | 95%(CI) | Sensitivity  (%) | Specificity  (%) | AUC | 95%(CI) | Sensitivity  (%) | Specificity  (%) |
| Random forest | COVID-19-F vs COVID-19-A | 0.950 | 0.860 - 1.000 | 0.9 | 0.9 | 0.976 | 0.951 - 1.000 | 0.950 | 0.900 |
|  | COVID-19-F vs HC | 1.000 | 1.000 - 1.000 | 1 | 1 | 1.000 | 1.000 - 1.000 | 1.000 | 1.000 |
| Support vector machine | COVID-19-F vs COVID-19-A | 0.987 | 0.881-1.000 | 1 | 1 | 0.926 | 0.861 - 0.992 | 0.950 | 0.860 |
|  | COVID-19-F vs HC | 1.00 | 1.000 - 1.000 | 1 | 1 | 0.927 | 0.858 - 0.995 | 0.830 | 1.000 |
| Logistic regression | COVID-19-F vs COVID-19-A | 0.927 | 0.526-1.000 | 1 | 0.9 | 0.770 | 0.680 - 0.868 | 0.670 | 0.860 |
|  | COVID-19-F vs HC | 0.980 | 0.778-1.000 | 1 | 1 | 0.834 | 0.752 - 0.916 | 0.680 | 1.000 |

AUC, area under the curve; CI, confidenceinterval; COVID-19-A, COVID-19 patients in acute phase; COVID-19-M, COVID-19 patients with mortality; HC, healthy control;
