# Supplementary material for: Dysregulated proteasome activity and steroid hormone biosynthesis are associated with mortality among patients with acute COVID-19
Source: J Transl Med. 2024 Jul 4;22:626. doi: 10.1186/s12967-024-05342-0 (PMC11229496; doi:10.1186/s12967-024-05342-0)
Supplement: Supplementary file 7 — Supplementary Material 7 [file 12967_2024_5342_MOESM7_ESM.docx]

Dear editor,

Thank you very much for handing the review of our manuscript (JTRM-D-24-02508) entitled “Dysregulated Proteasome Activity and Steroid Hormone Biosynthesis Are Associated with Mortality among Patients with Acute COVID-19”.

We have revised the manuscript according to the critical comments of the reviewers. We sincerely appreciate the improvement in our manuscript due to the comments of the reviewers. The changes are as follows:

***Reviewer #1****: In this paper the authors integrated proteomic and metabolomic analyses of plasma samples from a cohort of surviving hospitalized COVID-19 patients [acute COVID-19 (COVID-19-A) and recovered COVID-19 (COVID-19-R)], COVID-19 patients with mortality (COVID-19-M), other infectious disease controls (IDCs), and healthy controls (HCs). Machine learning models were constructed to identify specific patterns of COVID-19 and to discover acute-stage biomarkers that could predict mortality.*

*In overall the results obtained can be interesting but it is necessary that the authors improve the description and the discussion of their results. Some data are not clear and details are missing; this finding gives difficult the understanding of the data.*

**Reply:** Thank you so much for your comments. We sincerely appreciate the improvement of our manuscript due to these comments.

*Major concerns:*

1. *Why did the authors perform proteomics analysis only on 50 samples? Why did not they work on the same number of patients tested by metabolomics analysis?*

**Reply**: Thank you so much for your question. Samples from critically ill COVID-19 patients are extremely difficult to obtain. Therefore, we first collected 10 samples from each group for proteomic detection. Proteomic data analysis revealed significant metabolic enrichment in severely ill patients, so samples were subsequently collected partly for protein validation and partly for metabolomic analysis. Our systematic analysis provides a novel method for the early prediction of mortality in hospitalized COVID-19 patients. Moreover, typical molecular features were validated by ELISA in both the testing cohort and an independent validation cohort to ensure data reliability of proteomics.

2. *The authors should verify if the patients on which proteomics analysis has been performed reflect the same characteristics of the entire number of patients. They must perform statistical analysis to verify it.*

**Reply**: Thank you so much for your advice. To ensure data reliability, typical molecular features were validated by ELISA in the proteomics analysis cohort. Four inflammation-related proteins (CRP, SAA1, SAA2 and ORM1), three immunity-related proteins (IGHG1, IGLL5, and IGKV4-1), and four PSM proteins (PSMA1, PSMA6, PSMA7, and PSMB1) were selected for ELISA-based validation. These proteins were chosen based on the following screening criteria: high fold change and association with the proteomic features of COVID-19-A discussed above. The results have been supplied as below.

**Table. Verification of typical proteins in the proteomics analysis cohort.**

| **Proteins** | **group** | **n** | **min** | **max** | **median** | **mean** | **SD** |
| --- | --- | --- | --- | --- | --- | --- | --- |
| CRP ug/L | HC | 10 | 2988.9 | 17858.6 | 4671.2 | 6886.9 | 4821.1 |
| CRP ug/L | COVID-19-A | 10 | 30370.6 | 93274.4 | 63032.3 | 65016.0 | 22264.0 |
| CRP ug/L | COVID-19-R | 10 | 10116.5 | 55563.9 | 23030.3 | 26931.1 | 16616.3 |
| CRP ug/L | COVID-19-F | 10 | 13012.8 | 86013.7 | 34145.3 | 39294.7 | 25274.1 |
| CRP ug/L | IDC | 10 | 13899.5 | 57822.1 | 29771.7 | 31880.2 | 14114.6 |
| SAA1 ug/L | HC | 10 | 512.4 | 1520.2 | 1019.8 | 961.3 | 373.0 |
| SAA1 ug/L | COVID-19-A | 10 | 1550.9 | 4968.4 | 4482.5 | 3728.3 | 1341.5 |
| SAA1 ug/L | COVID-19-R | 10 | 1131.3 | 5112.1 | 2169.9 | 2524.9 | 1153.2 |
| SAA1 ug/L | COVID-19-F | 10 | 2967.5 | 5131.7 | 4293.1 | 4172.0 | 650.7 |
| SAA1 ug/L | IDC | 10 | 2259.8 | 4794.5 | 4014.7 | 3896.7 | 758.6 |
| SAA2 ug/L | HC | 10 | 105.1 | 268.6 | 166.8 | 172.5 | 43.1 |
| SAA2 ug/L | COVID-19-A | 10 | 125.8 | 469.7 | 253.0 | 264.6 | 103.8 |
| SAA2 ug/L | COVID-19-R | 10 | 79.9 | 323.4 | 180.6 | 188.7 | 83.9 |
| SAA2 ug/L | COVID-19-F | 10 | 86.6 | 350.2 | 151.4 | 165.8 | 71.9 |
| SAA2 ug/L | IDC | 10 | 130.7 | 479.5 | 192.6 | 211.7 | 100.0 |
| ORM1 ug/L | HC | 10 | 2007.7 | 5364.9 | 3695.8 | 3672.8 | 979.7 |
| ORM1 ug/L | COVID-19-A | 10 | 2565.8 | 13594.7 | 10094.3 | 9015.4 | 3739.9 |
| ORM1 ug/L | COVID-19-R | 10 | 1806.7 | 6709.2 | 4734.6 | 4383.4 | 1543.5 |
| ORM1 ug/L | COVID-19-F | 10 | 1833.6 | 7253.6 | 3447.3 | 3846.6 | 1937.6 |
| ORM1 ug/L | IDC | 10 | 1889.1 | 5364.1 | 2661.4 | 3025.7 | 1187.4 |
| IGHG1 pg/ml | HC | 10 | 2040.7 | 4655.1 | 3350.7 | 3341.4 | 844.3 |
| IGHG1 pg/ml | COVID-19-A | 10 | 975.6 | 3059.5 | 2138.3 | 2014.6 | 703.7 |
| IGHG1 pg/ml | COVID-19-R | 10 | 819.6 | 31745.6 | 1175.0 | 4300.5 | 9663.0 |
| IGHG1 pg/ml | COVID-19-F | 10 | 3970.5 | 26294.4 | 7200.4 | 11220.6 | 7505.0 |
| IGHG1 pg/ml | IDC | 10 | 3978.4 | 19185.3 | 5951.9 | 9291.5 | 6042.5 |
| IGLL5 ng/ml | HC | 10 | 99.2 | 223.6 | 150.8 | 154.1 | 43.3 |
| IGLL5 ng/ml | COVID-19-A | 10 | 48.9 | 106.1 | 64.6 | 69.8 | 20.0 |
| IGLL5 ng/ml | COVID-19-R | 10 | 45.2 | 104.7 | 67.7 | 71.1 | 18.6 |
| IGLL5 ng/ml | COVID-19-F | 10 | 74.7 | 234.7 | 129.6 | 140.6 | 52.9 |
| IGLL5 ng/ml | IDC | 10 | 90.3 | 164.8 | 109.9 | 116.6 | 25.9 |
| IGKV4-1 ng/ml | HC | 10 | 2.6 | 14.3 | 7.9 | 7.7 | 3.3 |
| IGKV4-1 ng/ml | COVID-19-A | 10 | 2.2 | 9.8 | 3.3 | 3.9 | 2.2 |
| IGKV4-1 ng/ml | COVID-19-R | 10 | 1.6 | 10.4 | 3.2 | 4.2 | 2.8 |
| IGKV4-1 ng/ml | COVID-19-F | 10 | 1.2 | 213.2 | 6.2 | 30.1 | 65.2 |
| IGKV4-1 ng/ml | IDC | 10 | 0.4 | 67.4 | 1.7 | 8.9 | 20.7 |
| PSMA1 ng/ml | HC | 10 | 28.1 | 101.7 | 89.0 | 80.0 | 23.3 |
| PSMA1 ng/ml | COVID-19-A | 10 | 70.4 | 150.7 | 105.9 | 106.6 | 23.6 |
| PSMA1 ng/ml | COVID-19-R | 10 | 32.7 | 174.8 | 101.9 | 104.1 | 37.7 |
| PSMA1 ng/ml | COVID-19-F | 10 | 100.4 | 201.0 | 143.6 | 145.0 | 30.3 |
| PSMA1 ng/ml | IDC | 10 | 65.2 | 224.8 | 126.7 | 127.5 | 43.9 |
| PSMA6 ng/ml | HC | 10 | 0.8 | 51.5 | 28.2 | 28.3 | 15.3 |
| PSMA6 ng/ml | COVID-19-A | 10 | 11.8 | 124.1 | 64.7 | 70.0 | 37.2 |
| PSMA6 ng/ml | COVID-19-R | 10 | 3.7 | 168.5 | 52.5 | 61.4 | 46.6 |
| PSMA6 ng/ml | COVID-19-F | 10 | 60.6 | 235.0 | 88.5 | 105.3 | 54.6 |
| PSMA6 ng/ml | IDC | 10 | 9.9 | 47.6 | 31.1 | 30.4 | 13.7 |
| PSMA7 ng/ml | HC | 10 | 17.1 | 24.0 | 20.5 | 20.3 | 2.5 |
| PSMA7 ng/ml | COVID-19-A | 10 | 27.5 | 50.2 | 36.2 | 36.4 | 7.6 |
| PSMA7 ng/ml | COVID-19-R | 10 | 28.7 | 51.6 | 34.2 | 37.2 | 8.0 |
| PSMA7 ng/ml | COVID-19-F | 10 | 35.4 | 89.1 | 49.5 | 51.9 | 14.2 |
| PSMA7 ng/ml | IDC | 10 | 33.4 | 62.8 | 45.3 | 46.7 | 8.0 |
| PSMB1 ng/ml | HC | 10 | 10.6 | 70.4 | 50.1 | 49.0 | 19.9 |
| PSMB1 ng/ml | COVID-19-A | 10 | 5.3 | 152.4 | 82.5 | 80.1 | 42.9 |
| PSMB1 ng/ml | COVID-19-R | 10 | 14.5 | 170.6 | 72.9 | 80.4 | 48.5 |
| PSMB1 ng/ml | COVID-19-F | 10 | 125.1 | 513.6 | 186.2 | 225.8 | 116.4 |
| PSMB1 ng/ml | IDC | 10 | 9.1 | 92.9 | 63.3 | 54.2 | 27.0 |

3. *In Results section the authors reported "To ensure data reliability, typical molecular features were validated in an independent test cohort." The authors must add data about this independent cohort in the manuscript. It is mandatory to insert a table with clinical characteristics of these patients, compare them with the training set and discuss these data.*

**Reply**: Thank you so much for your nice suggestion. It is really an important point. Accordingly, the clinical characteristics of patients in the training cohort and the independent testing cohort have been added to the **Supplementary Data 4.** Moreover, we compared the clinical characteristics of patients from these two cohorts. Moreover, the original **Supplementary Data 4** has been replaced to **Supplementary Data 5**.

**Supplementary Data 4: The clinical information of patients from training cohort and testing cohort.**

| **Characteristics** | **Training Cohort (n=30)** | | |  | **Testing Cohort (n=114)** | | | **P value ^a^** | **P value ^b^** | **P value ^c^** |
| --- | --- | --- | --- | --- | --- | --- | --- | --- | --- | --- |
|  | **COVID-19-A**  **(n=10)** | **COVID-19-M**  **(n=10)** | **HC**  **(n=10)** |  | **COVID-19-A**  **(n=42)** | **COVID-19-M**  **(n=41)** | **HC**  **(n=31)** |  |  |  |
| Gender (male/female) | 6/4 | 5/5 | 5/5 |  | 28/14 | 29/12 | 22/9 | 0.723 | 0.212 | 0.224 |
| Age (years) | 60.00  (54.50-64.25) | 69.50  (57.25-77.50) | 64.30  (56.00-69.00) |  | 78.00  (70.00-85.00) | 81.00  (72.00-87.00) | 71.24  (66.00-72.00) | <0.001 | 0.003 | 0.045 |
| Days of hospitalization | 13.00  (11.25-16.75) | 11.50  (3.50-26.75) | / |  | 10.00  (7.00-14.50) | 9.00  (4.00-15.50) | / | 0.089 | 0.643 | / |
| Critical score ^d^ | | | | | | | | | | |
| WBC, 10^9^/L | 5.69  (3.74-7.86) | 12.36  (8.82-16.78) | 6.16  (5.45-7.38) |  | 7.35  (4.88-9.02) | 9.73  (7.34-13.31) | 6.21  (5.04-7.32) | 0.207 | 0.081 | 0.531 |
| Neu, 10^9^/L | 3.85  (3.15-7.19) | 11.21  (8.32-15.84) | 3.57  (2.96-3.76) |  | 5.59  (3.34-7.65) | 8.59  (6.01-11.56) | 3.60  (2.44-4.19) | 0.335 | 0.021 | 0.893 |
| Lym, 10^9^/L | 0.58  (0.35-1.39) | 0.36  (0.28-0.46) | 1.85  (1.60-2.65) |  | 0.90  (0.47-1.32) | 0.56  (0.37-0.82) | 2.13  (1.68-2.56) | 0.458 | 0.082 | 0.925 |
| RBC, 10^12^/L | 4.30  (3.76-4.51) | 2.53  (2.32-3.65) | 4.86  (4.32-5.07) |  | 3.97  (3.42-4.36) | 3.92  (3.62-4.28) | 5.10  (4.76-5.24) | 0.190 | <0.001 | 0.196 |
| Hgb, g/L | 132.00  (113.50-135.50) | 83.50  (72.25-106.25) | 152.00  (134.00,157.50) |  | 123.50  (110.75-135.25) | 119.00  (112.00-128.00) | 153.00  (137.50-159.00) | 0.474 | <0.001 | 0.813 |
| Plt, 10^9^/L | 262.00  (146.50-344.50) | 84.50  (44.00-111.25) | 251.00  (222.00-278.50) |  | 207.50  (150.25-271.00) | 165.00  (129.00-215.00) | 263.00  (238.00-311.00) | 0.428 | 0.003 | 0.572 |
| Lac, mmol/L | 1.4  (1.15-1.58) | 2.05  (1.60-3.50) |  |  | 1.6  (1.15-2.25) | 2.10  (1.58-3.83) |  | 0.214 | 0.921 |  |
| PaO_2_/FiO_2_ | 258.62  (224.14-341.28) | 99.42  (73.77-291.76) |  |  | 227.42  (217.58-303.62) | 166.94  (109.33-217.24) |  | 0.872 | 0.293 |  |

^a^ P value between COVID-19-A in training cohort and testing cohort;

^b^ P value between COVID-19-M in training cohort and testing cohort COVID-19-M and HC;

^c^ P value between HC in training cohort and testing cohort;

^d^ Data are presented as median (percentiles).

COVID-19-A, COVID-19 patients in acute phase; COVID-19-M, COVID-19 patients with mortality; HC, healthy control; WBC, white blood cell; Neu, neutrophil; Lym, lymphocyte; RBC, red blood cell; Hgb: hemoglobin; Plt: platelet; Lac: lactic acid; PaO2/FiO2: oxygenation index.

*4. It should be useful if the authors add a figure with research plan and a clear table in the manuscript with the comparison between the clinical characteristics of survivors and non-survivors.*

**Reply**: Thank you so much for your suggestion. The research plan (**Figure 1**) and a clear table (**Supplementary Data 2**) has been added to the manuscript (Lines 207-271). We compared the clinical characteristics of survivors and non-survivors in **Supplementary Data 2**.

We performed a comprehensive molecular analysis of COVID-19-M patients, with the goal of predicting mortality during the acute phase of disease (**Fig. 1A**). COVID-19-M samples were collected from COVID-19 patients with mortality at the next day of hospitalization, as red box represents the samples collected from this group of patients (**Fig. 1B**). Omics analyses indicated that, other than immunosuppression, impaired steroid hormone biosynthesis, and elevated inflammation, excessive proteasome activity was the most prominent signature in the acute phase of disease among patients with mortality. Based on these data, we developed a new biomarker panel using machine learning algorithms to predict COVID-19 mortality among surviving patients during the acute phase of disease. This plasma biomarker panel was then validated in a larger independent cohort.

Moreover, the comparison between the clinical characteristics of survivors and non-survivors has been added to the **Supplementary Data 2.**

4. *The authors wrote that five machine learning classifiers (logistic regression, random forest, linear support vector machine, K-Nearest Neighbor, and decision tree) were used. They must insert in the manuscript details about the results obtained by these classifiers and explain because they selected the results by Random forest classification.*

**Reply**: Thank you so much for your advice. We sincerely checked the machine learning process. Eventually, three machine learning classifiers-linear support vector machine, random forest and logistic regression-were used to determine the best diagnostic model; accuracies and error rates were evaluated by 10-fold cross-validation (Lines 427-431). The diagnostic performances of these machine learning classifiers were expressed in **Supplemental Data 6**. Random forest classification identified as the best diagnostic model combination. It had AUC values of 0.950 and 1.000 for distinguishing COVID-19-M patients from COVID-19-A patients and HCs, respectively. It was able to distinguish COVID-19-M patients from COVID-19-A patients with 90.0% sensitivity and 90.0% specificity. Obviously, the diagnostic performance of linear support vector and logistic regression were lower than random forest classification.

K-Nearest Neighbor and decision tree are eventually excluded from the machine learning classifiers in our study. K-Nearest Neighbor is the spatial attribution of a certain category based on the similarity of variables. When K-Nearest Neighbor was used for variable training, the protein index had a large effect on the prediction of the whole model, which led to over-fitting and increased training errors. The decision tree is to judge the characteristic variables of each sample and assign the sample to the next node according to the judgment result. In this study, two proteins play a prominent role in sample allocation, which leads to inaccurate sample classification. Thus, K-Nearest Neighbor and decision tree are eventually excluded in our study.

**Supplementary Data 6. The diagnostic performance of these machine learning classifiers**

| Machine Learning | group | Training cohort | | | | Testing cohort | | | |
| --- | --- | --- | --- | --- | --- | --- | --- | --- | --- |
|  |  | AUC | 95%(CI) | Sensitivity  (%) | Specificity  (%) | AUC | 95%(CI) | Sensitivity  (%) | Specificity  (%) |
| Random forest | COVID-19-F vs COVID-19-A | 0.950 | 0.860 - 1.000 | 0.9 | 0.9 | 0.976 | 0.951 - 1.000 | 0.950 | 0.900 |
|  | COVID-19-F vs HC | 1.000 | 1.000 - 1.000 | 1 | 1 | 1.000 | 1.000 - 1.000 | 1.000 | 1.000 |
| Support vector machine | COVID-19-F vs COVID-19-A | 0.987 | 0.881-1.000 | 1 | 1 | 0.926 | 0.861 - 0.992 | 0.950 | 0.860 |
|  | COVID-19-F vs HC | 1.00 | 1.000 - 1.000 | 1 | 1 | 0.927 | 0.858 - 0.995 | 0.830 | 1.000 |
| Logistic regression | COVID-19-F vs COVID-19-A | 0.927 | 0.526-1.000 | 1 | 0.9 | 0.770 | 0.680 - 0.868 | 0.670 | 0.860 |
|  | COVID-19-F vs HC | 0.980 | 0.778-1.000 | 1 | 1 | 0.834 | 0.752 - 0.916 | 0.680 | 1.000 |

AUC, area under the curve; CI, confidence interval; COVID-19-A, COVID-19 patients in acute phase; COVID-19-M, COVID-19 patients with mortality; HC, healthy control.

6. *In the discussion section it is missing a comparison between the results obtained in this paper and previous studies focused on metabolomics studies on COVID19 patients. In literature, other papers identified biomarker predictors of COVID19 patients outcome. The authors should discuss the differences between their diagnostic model and those already published.*

**Reply**: This is a really nice point. The comparison between the results obtained in this paper and previous studies focused on metabolomics studies on COVID-19 patients has been added to the discussion section (Lines 499-517).

Metabolomics data analysis also revealed that the steroid hormone synthesis pathway is significantly inhibited in COVID-19 patients. Up to now, a large number of metabolomics studies have been carried out in COVID-19, and researchers have identified a variety of COVID-19-related metabolites, including multiple pathways such as glucose metabolism, urea cycle, and lipid metabolism. Ding Shi et al. verified the predictive ability of the combination of 7 metabolites on the severity of COVID-19 disease, including steroid substances, which was similar to our research results. It should be emphasized that previous studies focused on determining the severity of a patient's disease through metabolic markers. In this study, we mainly focus on the typical DEPs in the acute phase of the disease, which be able to predict the poor prognosis. Besides, it has been reported the close link between cytokine disorders in COVID-19 patients and certain metabolites such as choline and alpha-ketoglutaric acid, strongly suggesting potential therapeutic targets. Kaiming Wang et al. also revealed persistent inflammatory responses, platelet degranulation, and cell activation in multiple dysregulated metabolic pathways with in long COVID-19 patients.

***Reviewer #2****: The authors present a very advanced and well-designed proteomics/metabolomics study on COVID-19. However, reading the manuscript is a bit difficult and messy, especially for non-experts in the field of omics. It would be appropriate to review the drafting of the manuscript to guide the reader in viewing the results. Specific comments/suggestions follow:*

1. *please, review the punctuation; for example, line 25 "mortality. we applied";*

**Reply**: Thank you for your carefully review. We have corrected the error in line 25.

2. *supplementary data 1: the numbering of the data does not correspond to that cited in the text; the 52 patients reported in Suppl. data 1.3 are the patients surviving? Why is the number of days between collection and death reported? Instead, for Suppl. data 1.4 the days between collection and death are not reported, perhaps there was a data inversion? For Suppl. data 1.1 and 1.2 I would suggest replacing "clinic" with "demographic" in the title;*

**Reply**: Sorry for this unclear statement and we rearranged the layout of the **Supplementary Data 1** according to the manuscript. **Supplementary Data 1.1, 1.2, 1.3, 1.4** represent survival patient group (COVID-19-A/R), patients with mortality group (COVID-19-M), other infectious disease control group (IDC), and healthy control group (HC), respectively.

Totally, 155 plasma samples were collected from 103 patients with confirmed COVID-19 at Beijing Chao-Yang Hospital. Of these patients, 52 were discharged from the hospital and 51 died. Among the 52 discharged patients (survival patients), samples were collected in both the acute phase (1 day after admission) and the recovery phase (1–3 days before discharge, **Supplementary Data 1**.**1**). Thus, there were 104 samples in the **Supplementary Data 1.1** (original **Supplementary Data 1.3**). Sorry for this err description (*Why is the number of days between collection and death reported?*). The “Time from sample collection to death (d)” had been changed to “Time from sample collection to discharge (d)” (**Supplementary Data 1.1**, original **Supplementary Data 1.3**)

Time from sample collection to death (d) has been added to the **Supplementary Data 1.2** (original **Supplementary Data 1.4**).

Moreover, the “clinic” in the title in **Supplementary Data 1.3** and **1.4** (original Supplementary Data 1.1 and 1.2) has been replaced by "demographic".

3. *supplementary data 2: it would be appropriate to indicate with a superscript letter the values that are significantly different compared to another group: e.g. a p < 0.05 versus IDC, b p < 0.05 versus HC;*

**Reply**: Thank you for your nice advice. We have refined the content of **Supplementary Data 2** to clarify the differences between each group.

**Supplementary Data 2: The clinical information and conducted biochemical laboratory tests.**

| Gender (male/female) | 37/14 | 28/13 | 34/18 | 33/18 | 0.733 | 0.931 |
| --- | --- | --- | --- | --- | --- | --- |
| Age (years) | 63.00(52.00-70.00) | 73.00(55.00-78.00) | 75.00(65.00-83.75) ^cd^ | 77.00(69.00-86.00) ^g^ | 0.345 | 0.743 |
| Days of hospitalization | 14.00(7.75- 24.25) | / | 11.50(7.25-14.75) | 10.00(4.00-18.00) | 0.137 | 0.121 |
| Critical score ^h^ | | | | | | |
| WBC, 10^9^/L | 7.67(4.66-15.86) ^e^ | 6.19(5.07-7.26) | 7.18(4.64-8.63) | 9.99(7.76-13.67) ^fg^ | 0.047 | <0.001 |
| Neu, 10^9^/L | 5.78(3.16-13.39) ^e^ | 3.59 (2.52-4.09) | 5.09(3.21-7.61) ^d^ | 8.93(6.08-11.89) ^fg^ | <0.001 | <0.001 |
| Lym, 10^9^/L | 0.99(0.48-1.55) ^e^ | 2.11(1.65-2.58) | 0.89(0.43-1.31) ^d^ | 0.48(0.36-0.75) ^fg^ | <0.001 | <0.001 |
| RBC, 10^12^/L | 3.97(3.31-4.37) ^e^ | 5.01(4.50-5.22) | 4.01(3.63-4.41) ^d^ | 3.77(3.29-4.19) ^g^ | <0.001 | <0.001 |
| Hgb, g/L | 120.50(100.00-130.00) ^e^ | 152.50(135.50-158.25) | 124.00(111.00-135.00) ^d^ | 117.00(105.00-126.00) ^g^ | <0.001 | <0.001 |
| Plt, 10^9^/L | 223.00(166.75-331.75) | 257.00(237.00-299.25) | 218.00(151.00-284.00) ^d^ | 151.00(103.00-213.00) ^fg^ | 0.027 | <0.001 |
| Lac, mmo/L | / | / | 1.5(1.15-2.1) | 2.10(1.58-3.73) | / | <0.001 |
| PaO_2_/FiO_2_ | 279.31(165.43-348.60) | / | 272.41(224.14-304.48) | 149.52(99.31-217.24) | 0.890 | <0.001 |
| Gender (male/female) | 37/14 | 28/13 | 34/18 | 33/18 | 0.733 | 0.931 |

^a^ P value among IDC, HC and surviving COVID-19;

^b^ P value among HC, surviving COVID-19 and COVID-19 patients with mortality;

^c^ P value between Surviving COVID-19 and IDC;

^d^ P value between Surviving COVID-19 and HC;

^e^ P value between Surviving IDC and HC;

^f^ P value between COVID-19 patients with mortality and Surviving COVID-19;

^g^ P value between COVID-19 patients with mortality and HC;

^h^ Data are presented as median (percentiles);

COVID-19-A/R, surviving COVID-19 patients; COVID-19-M, COVID-19 patients with mortality; IDC,other infectious disease control; HC, healthy control; WBC, white blood cell; Neu, neutrophil; Lym, lymphocyte; RBC, red blood cell; Hgb: hemoglobin; Plt: platelet; Lac: lactic acid; PaO_2_/FiO_2_: oxygenation index.

4. *line 249: Fig. 2A and Fig. 2B are reversed in the text, please correct;*

**Reply**: Thank you for your nice advice. We have switched the expression of Fig. 2A and Fig. 2B (Lines 248-252).

*5. lines 270-272: for cluster 4, in the IDC group the DEPs appear even lower;*

**Reply**: Thank you so much for the nice question. In clinical practice, co-infections

are common in patients with pneumonia. To find out the unique characteristics of the COVID-19, HC and IDC were both used as controls in this study. In cluster 4, the expression of these DEPs in COVID-19-A/COVID-19-R groups were higher than that in IDC group, implying that the expression of these DEPs might be more susceptible to other pathogens (Lines 273-275).

*6. line 277: as observed in Fig. 2C, "**increased" instead of "decreased"?*

**Reply**: Sorry for this error description. The “decreased” has been changed to “increased” (Line 283) *.*

*7. line 285: from figure 2G the correlation appears negative;*

**Reply**: Sorry for this error description. The levels of proteins in cluster 3 were negatively correlated with Lym and WBC counts (Line 292).

*8. lines 302-304: the description of cluster 5 is missing;*

**Reply**: Thank you so much for your question. The description of cluster 5 has combined with cluster 2. As shown in line 310, three distinct metabolite expression patterns across patients with different phases of disease were observed, including two decreasing clusters (1 and 4), an increasing cluster (2 and 5), and an inverted “V” cluster (3) (Fig. 3C).

*9. Fig. 4B: please, correct the title.*

**Reply**: Sorry for the error spelling. “Meatbolomic” has been corrected to “Metabolomic”.
